# Characterization of *WRKY *co-regulatory networks in rice and Arabidopsis

**DOI:** 10.1186/1471-2229-9-120

**Published:** 2009-09-22

**Authors:** Stefano Berri, Pamela Abbruscato, Odile Faivre-Rampant, Ana CM Brasileiro, Irene Fumasoni, Kouji Satoh, Shoshi Kikuchi, Luca Mizzi, Piero Morandini, Mario Enrico Pè, Pietro Piffanelli

**Affiliations:** 1 Department of Biomolecular Sciences and Biotechnology, University of Milan, via Celoria 26, 20133 Milan, Italy; 2 School of Computing, University of Leeds, LS2 9JT Leeds, UK; 3 Rice Genomics Unit, Parco Tecnologico Padano, via Einstein, 26900 Lodi, Italy; 4 UMR BGPI, CIRAD, Campus International de Baillarguet, 34398 Montpellier Cedex 5, France; 5 Parque Estação Biológica, Embrapa Recursos Genéticos e Biotecnologia, Av. W5 Norte, 02372, Brasília DF, Brazil; 6 UMR DAP, CIRAD, Avenue Agropolis, 34398 Montpellier Cedex 5, France; 7 Department of Molecular Genetics, National Institute of Agrobiological Sciences, 2-1-2 Kannon-dai, Tsukuba, Ibaraki 305-8602, Japan; 8 Department of Biology, University of Milan and CNR Institut of Biophysics (Milan Section), via Celoria 26, 20133 Milan, Italy; 9 Sant'Anna School for Advanced Studies, Piazza Martiri della Libertà 33, 56127 Pisa, Italy

## Abstract

**Background:**

The WRKY transcription factor gene family has a very ancient origin and has undergone extensive duplications in the plant kingdom. Several studies have pointed out their involvement in a range of biological processes, revealing that a large number of *WRKY *genes are transcriptionally regulated under conditions of biotic and/or abiotic stress. To investigate the existence of *WRKY *co-regulatory networks in plants, a whole gene family *WRKY*s expression study was carried out in rice (*Oryza sativa*). This analysis was extended to *Arabidopsis thaliana *taking advantage of an extensive repository of gene expression data.

**Results:**

The presented results suggested that 24 members of the rice *WRKY *gene family (22% of the total) were differentially-regulated in response to at least one of the stress conditions tested. We defined the existence of nine Os*WRKY *gene clusters comprising both phylogenetically related and unrelated genes that were significantly co-expressed, suggesting that specific sets of *WRKY *genes might act in co-regulatory networks. This hypothesis was tested by Pearson Correlation Coefficient analysis of the Arabidopsis *WRKY *gene family in a large set of Affymetrix microarray experiments. *AtWRKYs *were found to belong to two main co-regulatory networks (COR-A, COR-B) and two smaller ones (COR-C and COR-D), all including genes belonging to distinct phylogenetic groups. The COR-A network contained several *AtWRKY *genes known to be involved mostly in response to pathogens, whose physical and/or genetic interaction was experimentally proven. We also showed that specific co-regulatory networks were conserved between the two model species by identifying Arabidopsis orthologs of the co-expressed *OsWRKY *genes.

**Conclusion:**

In this work we identified sets of co-expressed *WRKY *genes in both rice and Arabidopsis that are functionally likely to cooperate in the same signal transduction pathways. We propose that, making use of data from co-regulatory networks, it is possible to highlight novel clusters of plant genes contributing to the same biological processes or signal transduction pathways. Our approach will contribute to unveil gene cooperation pathways not yet identified by classical genetic analyses. This information will open new routes contributing to the dissection of WRKY signal transduction pathways in plants.

## Background

*WRKY *genes code for transcription factors characterized by the presence of one or two 60 amino-acid WRKY motif including a very highly-conserved WRKYGQK sequence together with a zinc-finger-like motif CX_4-7 _-CX_23-28 _-HX_1-2 _-(H/C) that provides binding properties to DNA. Most of the WRKY proteins bind to the conserved W-box (C/T)TGAC(T/C) [1-4]. The *WRKY *genes were initially believed to be plant-specfic [5], but their ancient origin, is witnessed by the presence of two-domain WRKY in two non-photosynthetic unicellular Eukaryota organisms: in the Diplomonadida *Giardia lamblia *and in the Mycetozoa *Dictyostelium discoideum*. An ancestor *WRKY *gene may, therefore, have already been present before divergence of animals, fungi and plants, but was probably lost in the former groups [6]. The *WRKY *genes have experienced an incredible evolutionary success in the plant kingdom where successive duplication events have resulted in large gene families that includes up to 74 members in Arabidopsis and over one hundred in rice. The first record of a *WRKY *gene [7] came from cloning genes from sweet potato (*Ipomoea batatas*) followed by the description of two *WRKY *genes (*ABF1 *and *ABF2*) in wheat, barley and wild oat [8]. Eulgem *et al*. [9] described most of the Arabidopsis *WRKY *genes and classified them on the basis of both the number of WRKY domains and the features of their zinc-finger-like motif. WRKY proteins with two WRKY domains belong to group 1, whereas most proteins with one WRKY domain belong to group 2. In general, the WRKY domains of group 1 and group 2 members have the same type of zinc finger motif, whose pattern of potential zinc ligands CX_4-5 _-CX_22-23 _-HXH is unique among all known zinc-finger-like motifs. The single zinc finger motif of a small subset of WRKY proteins is distinct from that of group 1 and 2 members. Instead of a C_2_H_2 _pattern, their WRKY domains contain a C_2_HC motif. As a result of this distinction, they were assigned to group 3 [9].

Several studies have shown that *WRKY *genes are involved in many different biological processes such as response to wounding [10], senescence [4,11], development [12] dormancy and drought tolerance [13], solar ultraviolet-B radiation [14], metabolism [15,16], hormone signalling pathways [17,18] and cold [19]. However, numerous *WRKY *genes are involved in response to biotic stress and pathogen attacks. The first evidence for this was shown by Rushton *et al*. [20] who found three *WRKY *genes that specifically were able to bind to three W-box in the promoter of the pathogenesis-related gene *PR1 *in parsley. Later studies showed the involvement of other *WRKY *genes in response to pathogen, either because they are regulated during infection [3,21-24] or due to their proximity to well characterized genes that play a crucial role in plant defence, such as *NPR1 *in Arabidopsis [2,25].

Although there are several publications describing *WRKY *genes, only a few of the respective mutants show a clear link between a *WRKY *gene and an altered phenotype. In Arabidopsis, the gene *TRANSPARENT TESTA GLABRA2 *(*TTG2*) encodes a WRKY transcription factor (*AtWRKY44*) that, when mutated, causes disruptions to trichome development, different seed coat colour and mucilage production [12]. A second WRKY transcription factor of Arabidopsis is involved in seed development (*AtWRKY10*, encoded by *MINISEED3 *[26]); the corresponding mutants show smaller seeds and early cellularization of the endosperm. Despite the availability of insertion mutants for nearly every gene in Arabidopsis [27], a reverse genetic approach has so far only succeeded in revealing pathogen-related phenotypes for a few *WRKY *genes; the observed phenotypes were often weak or described as "enhanced susceptibility" [28,29]. Typically, phenotypes become detectable by combining mutants in multiple *WRKY *genes or by over-expression analyses [25]. There are a few exceptions: the atypical gene *AtWRKY52 *that provides resistance to *Ralstonia solanacearum *[30], *AtWRKY70 *whose mutant shows enhanced susceptibility to *Erysiphe cichoracearum *and differential accumulation of anthocyanins following methyl jasmonate application [31,32]. Similarly, mutation of *AtWRKY33 *results in enhanced susceptibility to two necrotrophic pathogens, namely *Botrytis cinerea *and *Alternaria brassicola *[33]. For 20 *WRKY *insertion mutants in rice screened in our laboratory (data unpublished) no phenotypic variation was observed for host and non-host pathogen interaction.

The most frequent hypothesis to explain the lack of phenotype in knockout plants is functional redundancy [25,28]. Indeed, lines in which multiple *WRKY *genes were knocked out, are often produced to test whether a small group of phylogenetically-related genes are redundantly involved in a certain function. It is, therefore, important to clearly understand the phylogenetic relationships between genes of the same family. This has been extensively performed for *WRKY *genes both in rice and Arabidopsis [9,34,35]. This strategy has been successful in some cases [22,28], but it is still insufficient to pinpoint genes that might be part of the same regulatory network. Another possible explanation for the lack of a clear association between *WRKY *genes and a specific phenotype was proposed by Ülker and Somssich [6] who demonstrated that in parsley several WRKY transcription factors, by binding to W-box in the same promoter, are involved in regulating expression of one or more target genes. To understand the function of a single *WRKY *gene it is crucial to identify all the genes participating in the associated regulatory network. In the first attempt to unveil the network of *WRKY *genes involved in pathogen response using a microarray approach, Wang *et al*. [29] identified five *WRKY *genes (belonging to three different phylogenetic subgroups) involved in systemic acquired resistance.

To identify the *OsWRKY *genes involved in response to *Magnaporthe *infection and osmotic stress, and to ascertain the existence of co-expression gene clusters, a custom *WRKY *specific oligo array was designed. Hybridisation results highlighted the involvement of *OsWRKY *genes that were differentially regulated in conditions of biotic and/or osmotic stress. Some of these genes were co-expressed, suggesting a possible co-regulation in the same signal transduction pathways. We also performed a Pearson Correlation Coefficient (PCC) analysis using public Arabidopsis Affymetrix expression data, which is the largest and most reliable transcriptome dataset available. Two main co-regulatory networks were identified, one of which contains many of the *AtWRKY *genes known to be involved in response to pathogens. The different sets of co-expressed *WRKY *genes described in rice and Arabidopsis contained a significant number of phylogenetically distantly-related genes. The power of the described approach was validated by the Pearson Correlation analysis of the *MADS-BOX *genes which correctly identified most members shown to belong to the major network controlling floral patterning and differentiation. Our results revealed the usefulness of characterizing co-regulatory networks to identify potential novel candidate genes cooperating in the same biological processes or signal transduction pathways. These candidates will, then, need to be experimentally tested at the functional level.

## Results

WRKY proteins have been previously studied in a wide range of plant species [5,8,16,19,36] and shown to be involved in the regulation of several cellular processes, such as control of metabolic pathways, drought, heat shock, senescence, development and hormone signalling. However, the most studied role of this gene family appears to be in response to biotic and abiotic stress stimuli. The main goal of the work presented here was to perform a whole gene family transcription analysis of the rice and Arabidopsis *WRKY*s to identify those that are co-expressed in biotic and abiotic stress responses and that are potentially part of common signal transduction co-regulatory networks.

### Phylogenetic analyses of rice WRKY gene family

**Figure 1:**
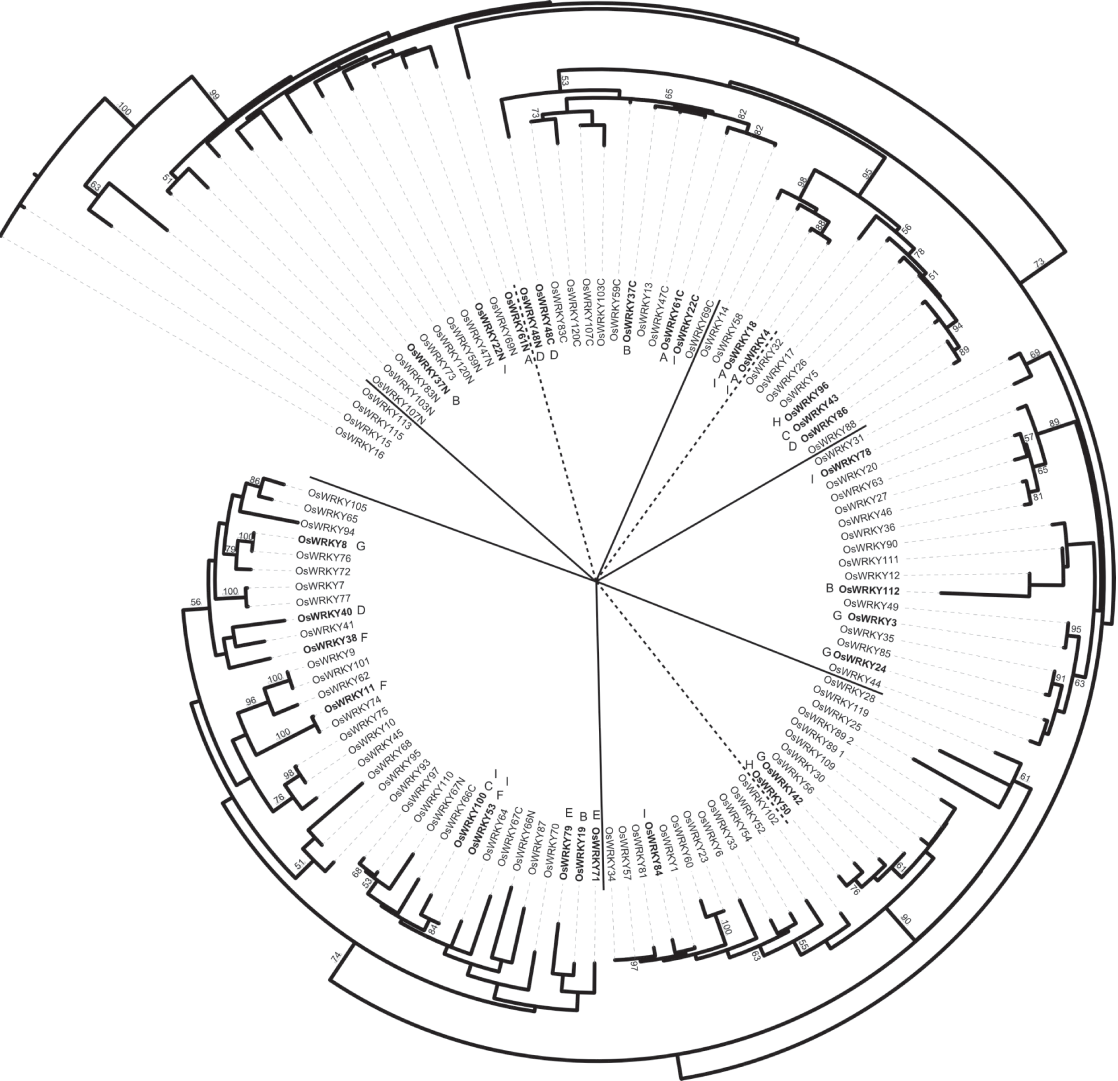
**Phylogenetic tree of rice OsWRKY whole gene family**. Phylogenetic tree of rice WRKY proteins. The tree was obtained on the basis of WRKY domain sequences of the 104 rice WRKY protein sequences with the Maximum Likelihood method using PHYML [68]. Both the N and the C WRKY domains were considered for those proteins bearing two domains. Bootstrap values higher than 50 are indicated in the nodes. Letters indicate the nine clusters of co-expressed genes, as presented in Figure 2 and Figure 3. The tree image was produced using iTOL software [69].

One hundred and four *WRKY *genes were identified in the rice genome by searching TIGR release 5 database using the PFAM ID PF03106 and Genbank using tblastn with the consensus WRKY domain as the query sequence (see Methods). Manual inspection of the results obtained was performed to eliminate duplicated entries [see Additional file 1]. Phylogenetic analysis performed with the Maximum Likelihood method using all 104 proteins containing a single or double WRKY domain, divided the genes into 5 main phylogenetic groups (Figure 1). Additional sub-groups and smaller clades were identified within each group, based upon bootstrap values. The *OsWRKY *genes containing two domains (see OsWRKY names ending with N and C) represented two distinct clades of the same phylogenetic main group (see Figure 1). Bootstrap values of some nodes of the tree were found to be moderately low; this finding in the global OsWRKY analysis was not completely unexpected due to the low degree of conservation, short length of the WRKY domain and to the large size of the *OsWRKY *gene family. To attempt to improve the bootstrap values it would be necessary to align longer sequence stretches, but this approach would not be of help for *WRKY *genes as, outside the WRKY domain, amino acid sequences are poorly conserved.

To reconstruct the evolutionary relationships of *WRKY *genes in rice and Arabidopsis, a phylogenetic tree was built using all of the WRKY domain sequences from the two species. Our analysis is in good agreement with the classification reported by Eugelm *et al*. [9] in Arabidopsis [see Additional file 2]. The Os-AtWRKY tree obtained in this study suggests a further division of group 3 into three distinct sub-groups: 3A, 3B, 3C [see Additional file 2]. More precisely, the presence of a sub-group containing only Arabidopsis *WRKY *genes (3A) was observed, a second one including only *OsWRKY *genes (named 3C) and a third one (3B) containing the remaining genes. This partition is likely to be the consequence of a series of species-specific duplication events in the *OsWRKY *3 group, which occurred after the separation of Monocotyledons from Dicotyledons [35] and that are well documented in rice [18,37]. These events led to the great expansion of the rice WRKY group 3, to a total of 36 genes which represent 35% of the *OsWRKY *gene family.

### Rice WRKY whole gene family transcriptome analysis

**Figure 2:**
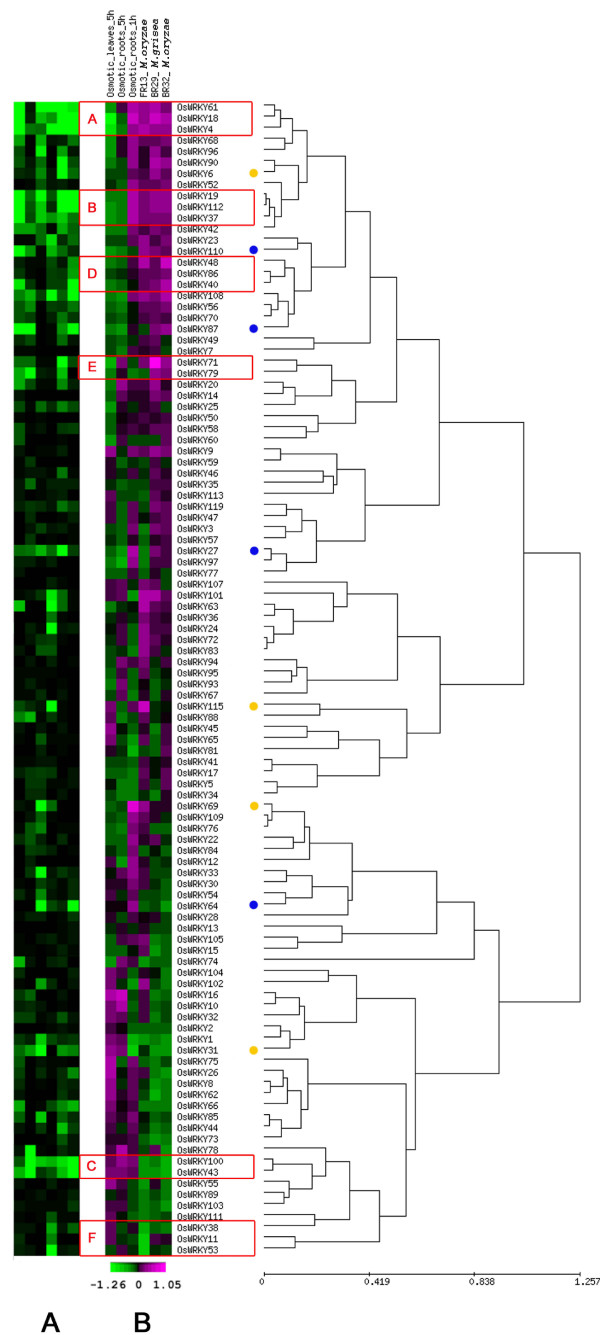
**Clustering of *OsWRKY *genes according to their expression profiles in the OsWRKYARRAY**. The OsWRKYARRAY was constitued of 104 probesets representing all members of the rice *WRKY *gene family. The expression of the 104 *OsWRKY *genes was assessed upon inoculation with *Magnaporthe oryzae *isolate from rice (FR13), *M. oryzae *BR32 from wheat, *M. grisea *BR29 from crabgrass and upon application of osmotic stress (mannitol) in hydroponic conditions. **Panel A **T-test P-values (shown by a green - black gradient) of treated vs control of the corresponding ratios shown in Panel B. The range of log transformed P-values comprised values between 0.01 (green) and 1 (black). P-values lower than 0.01 were visualized as 0.01. **Panel B **log_2_(Treated/Control) ratio values (shown by a green - magenta gradient). Red boxes with capital letters from A to F highlight the presence of co-expressed *WRKY *gene clusters. A blue dot indicates a *OsWRKY *gene differentially-regulated in two different stress conditions; a yellow dot indicates a *OsWRKY *gene-differentially regulated only in one stress condition. See Table 1 for numeric values of differentially-regulated *OsWRKY *genes.

A custom 60-mer oligo array (OsWRKYARRAY) was developed for rice *WRKY *gene family transcriptome analysis. This array contained the complete set of *OsWRKY *gene-specific probes based upon the hundred and four known genomic sequences [see Additional file 3]. RNA samples isolated from leaves and roots of two week-old rice plants following biotic or abiotic stress treatments were used for hybridisation on the OsWRKYARRAY. The expression of the 104 *OsWRKY *genes was assessed in the following conditions: 1) upon inoculation of leaves with one *Magnaporthe oryzae *isolate from rice, FR13, and two non-rice *Magnaporthe *isolates, *M. oryzae *BR32 from wheat and *M. grisea *BR29 from crabgrass; 2) upon application of osmotic stress in hydroponic conditions. Considering that fungal appressoria take about 16 hours to penetrate a rice leaf epidermal cell [38], leaf samples were collected 24 hours post inoculation (hpi) with the three *Magnaporthe *strains. The aim of this experiment was to assess early rice responses to fungal infection. RNA purified for these experiments came from the same batch of rice plants used for the cytological and molecular characterization of rice-*Magnaporthe *interactions described in Faivre-Rampant *et al*. [39]. For the study of *OsWRKY *gene expression upon osmotic stress conditions, samples were collected 1 hour (roots) and 5 hours (leaves and roots) after osmotic treatment. Gene expression results obtained from OsWRKYARRAY hybridisation experiments are reported in Table 1 and Figure 2. *OsWRKY *genes were considered to be up or down regulated when the logarithm values of the ratio of expression levels between treated and control RNA were higher than 0.2 or lower than -0.2 with the associated corrected P-value < 0.05. The analysis of differentially expressed *OsWRKY *genes revealed that 24 (22% of the total) were differentially regulated (down or up) in at least one of the six tested stress conditions (Table 1). Interestingly, among these 24 rice *WRKY *genes, gene expression of eight (*OsWRKY4*, *OsWRKY18*, *OsWRKY61*, *OsWRKY19*, *OsWRKY37*, *OsWRKY112, OsWRKY43 *and *OsWRKY100*) changed in response to both biotic and osmotic stress stimuli (in bold in Table 1). A few genes appeared to be differentially regulated only in a limited number of stress conditions, such as *OsWRKY110, OsWRKY87, OsWRKY27, OsWRKY64 *(see blue dots in Figure 2). *OsWRKY110 *was induced by FR13 infection, but repressed upon osmotic stress in leaves. *OsWRKY87 *was up regulated by BR32, whereas it was down regulated at late stage in both osmotic-stressed roots and leaves. *OsWRKY27 *is up regulated by BR29 and upon osmotic stress, but only in roots at 1 hpi. Finally *OsWRKY64 *was repressed by BR32 and induced only in roots by osmotic stimuli at an early stage. In addition, four genes *OsWRKY6*, *OsWRKY115, OsWRKY69 *and *OsWRKY31 *were differentially-regulated only in one stress condition (see yellow dots in Figure 2).

**Table 1:** List of differentially regulated Os*WRKY *genes upon pathogen and osmotic stress

**Trm**	**Name**	**R**	**M**	**A**	**P. Value**	**Trm**	**Name**	**R**	**M**	**A**	**P. Value**
***M. grisea *crabgrass (BR29)**	***OsWRKY18***	**1.547**	**0.629**	**8.684**	**3.42E-06**	**Leaves Osmotic 5 hours**	***OsWRKY4***	**0.569**	**-0.813**	**9.319**	**5.11E-10**
			
	***OsWRKY61***	**1.426**	**0.511**	**10.493**	**2.56E-05**		***OsWRKY18***	**0.419**	**-1.256**	**8.771**	**1.13E-08**
			
	***OsWRKY4***	**1.329**	**0.410**	**9.385**	**7.82E-05**		***OsWRKY61***	**0.736**	**-0.443**	**10.770**	**3.46E-05**
			
	*OsWRKY71*	2.075	1.053	7.332	8.35E-04		***OsWRKY37***	**0.777**	**-0.364**	**11.870**	**1.99E-03**
			
	***OsWRKY19***	**1.304**	**0.383**	**11.830**	**8.74E-04**		*OsWRKY87*	0.807	-0.309	9.781	2.90E-03
			
	***OsWRKY112***	**1.326**	**0.407**	**12.200**	**1.57E-03**		***OsWRKY19***	**0.832**	**-0.265**	**12.135**	**2.90E-03**
			
	*OsWRKY27*	1.208	0.273	12.453	1.57E-03		***OsWRKY112***	**0.756**	**-0.404**	**12.640**	**5.02E-03**
			
	*OsWRKY6*	1.251	0.323	10.882	3.24E-03		*OsWRKY110*	0.787	-0.346	7.642	8.27E-03
			
	*OsWRKY90*	1.408	0.494	7.442	8.87E-03		*OsWRKY40*	0.842	-0.248	9.187	2.42E-02
			
	***OsWRKY100***	**0.816**	**-0.294**	**12.756**	**3.15E-02**		*OsWRKY63*	0.850	-0.234	8.670	4.97E-02
			
	***OsWRKY37***	**1.180**	**0.238**	**11.520**	**4.13E-02**		*OsWRKY43*	1.206	0.270	11.571	7.24E-02
			
	*OsWRKY44*	0.815	-0.296	7.956	4.67E-02		*OsWRKY20*	0.812	-0.300	8.904	7.46E-02
			
	***OsWRKY43***	**0.799**	**-0.324**	**11.214**	**4.76E-02**		*OsWRKY14*	0.874	-0.194	9.003	7.63E-02
			
	*OsWRKY20*	1.367	0.451	8.931	5.06E-02		*OsWRKY42*	0.864	-0.211	10.513	8.57E-02
	
	*OsWRKY42*	1.193	0.255	10.249	6.45E-02	**Roots Osmotic 1 hour**	*OsWRKY64*	1.269	0.343	14.766	7.97E-04
			
	*OsWRKY96*	1.301	0.380	6.908	8.05E-02		***OsWRKY19***	**1.469**	**0.555**	**11.659**	**1.67E-03**
		
***M. oryzae *wheat (BR32)**	***OsWRKY18***	**1.468**	**0.554**	**9.011**	**2.68E-05**		*OsWRKY31*	0.606	-0.723	8.649	6.29E-03
			
	*OsWRKY40*	1.396	0.481	9.326	2.68E-05		*OsWRKY69*	1.799	0.847	8.030	6.86E-03
			
	***OsWRKY4***	**1.360**	**0.444**	**9.563**	**8.05E-05**		***OsWRKY61***	**1.512**	**0.597**	**10.079**	**1.14E-02**
			
	*OsWRKY108*	1.399	0.485	8.593	8.05E-05		*OsWRKY33*	1.266	0.340	11.102	1.49E-02
			
	***OsWRKY100***	**0.707**	**-0.500**	**12.407**	**1.38E-03**		*OsWRKY96*	1.350	0.433	8.881	1.66E-02
			
	*OsWRKY87*	1.253	0.325	9.493	1.38E-03		***OsWRKY112***	**1.491**	**0.577**	**12.354**	**3.81E-02**
			
	***OsWRKY43***	**0.705**	**-0.505**	**11.178**	**1.38E-03**		*OsWRKY85*	1.157	0.210	10.115	3.81E-02
			
	*OsWRKY64*	0.777	-0.365	13.627	1.38E-03		***OsWRKY37***	**1.373**	**0.458**	**11.500**	**3.81E-02**
			
	***OsWRKY19***	**1.245**	**0.316**	**11.556**	**4.24E-03**		*OsWRKY1*	0.725	-0.464	7.044	3.81E-02
			
	***OsWRKY112***	**1.345**	**0.427**	**11.932**	**5.89E-03**		***OsWRKY100***	**1.206**	**0.271**	**12.977**	**3.81E-02**
			
	***OsWRKY61***	**1.177**	**0.235**	**10.309**	**1.66E-02**		***OsWRKY18***	**1.543**	**0.626**	**8.633**	**4.54E-02**
		
***M. oryzae *rice (FR13)**	***OsWRKY4***	**1.471**	**0.557**	**10.546**	**8.26E-05**		*OsWRKY27*	1.406	0.492	12.147	4.87E-02
	
	*OsWRKY53*	0.627	-0.673	9.566	7.05E-04	**Roots Osmotic 5 hours**	***OsWRKY100***	**1.330**	**0.411**	**13.325**	**3.08E-03**
			
	*OsWRKY108*	1.323	0.404	9.194	8.20E-04		*OsWRKY87*	0.777	-0.364	9.850	3.08E-03
			
	*OsWRKY115*	1.591	0.670	9.389	8.20E-04		*OsWRKY78*	1.386	0.471	7.519	4.89E-03
			
	*OsWRKY63*	1.453	0.539	9.751	1.10E-03		***OsWRKY43***	**1.181**	**0.240**	**11.126**	**1.60E-02**
			
	***OsWRKY61***	**1.321**	**0.402**	**11.260**	**2.68E-03**		*OsWRKY20*	1.340	0.423	9.206	2.40E-02
			
	*OsWRKY24*	1.346	0.429	10.918	4.53E-03						
			
	*OsWRKY23*	1.285	0.361	9.387	2.11E-02						
			
	*OsWRKY101*	1.420	0.506	6.734	2.11E-02						
			
	*OsWRKY110*	1.363	0.447	8.469	6.05E-02						
			
	*OsWRKY100*	0.757	-0.402	13.468	6.65E-02						
			
	*OsWRKY38*	0.713	-0.488	7.540	6.92E-02						

List of *OsWRKY *genes differentially-expressed in the tested experimental conditions with a corrected (False discovery rate) P-value < 0.05 (grey entries indicate a P-value < 0.1). **Trm **indicated the applied stress treatment. **R **indicates the ratio of expression levels between treated and control RNA samples. **M **indicates log_2_(R). **A **indicates log_2 _of the average intensity signal from microarray experiment among technical and biological replicates. Genes highlighted in bold are differentially-regulated in three or more experimental conditions.

Clustering analysis of the data obtained with the OsWRKYARRAY was performed to pinpoint genes with similar expression profiles between different stress conditions. This analysis highlighted the following points (see red boxes in Figure 2):

i) three clusters of genes co-expressed in all test conditions for biotic and osmotic stress. In cluster A (*OsWRKY4*, *OsWRKY18*, *OsWRKY61*) and B (*OsWRKY19*, *OsWRKY37*, *OsWRKY112*) genes are up regulated after infection with all 3 *Magnaporthe *strains, but repressed upon osmotic stress treatment, in leaves and in roots. In contrast, in the small cluster C, genes *OsWRKY100 *and *OsWRKY43 *are down regulated after *Magnaporthe *interactions, but induced in roots and leaves after osmotic stress stimuli.

ii) three clusters of genes differentially expressed specifically upon one *Magnaporthe *interaction. Genes *OsWRKY48*, *OsWRKY86 *and *OsWRKY40 *(Cluster D) are induced after infection with *M. oryza*e BR32, while *OsWRKY71 *and *OsWRKY79 *(Cluster E) with *M. grisea *BR29. The remaining cluster F includes *OsWRKY38*, *OsWRKY11 *and *OsWRKY53 *genes, which are down regulated by *Magnaporthe oryzae *strain FR13.

**Figure 3:**
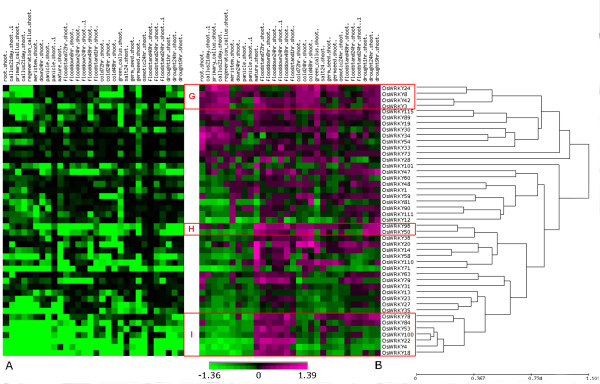
**Clustering of *OsWRKY *genes according to their expression profile in the NIAS 22 K array**. Clustering of the 50 *OsWRKY *genes present in the NIAS 22 K array according to their expression profiles in 30 experiments (upon abiotic stress conditions and in different plant tissues) was performed. **Panel A **T-test P-values (shown by a green - black gradient) of treated vs control of the corresponding ratios shown in Panel B. The range of log transformed P-values comprised values between 0.01 (green) and 1 (black). P-values lower than 0.01 were visualized as 0.01. **Panel B **log_2_(Treated/Control) ratio values (shown by a green - magenta gradient). Red boxes with capital letters from G to I highlight the presence of co-expressed *WRKY *gene clusters.

To broaden the *WRKY *gene family expression profile obtained with the OsWRKYARRAY, *WRKY *expression data from the 22 K NIAS array (National Institute of Agrobiological Sciences) were extracted to highlight those genes that are co-expressed in a wider range of abiotic stress conditions, as well as at different developmental stages (shoot, meristem, panicle). Since in the 22 K NIAS array, only a subset of 50 *WRKY *genes is present (out of 104 of the whole gene family), a separate clustering analysis was performed (Figure 3). The gene expression data analysis was carried out using the same rationale as was applied to the OsWRKYARRAY (logarithm values of the ratio higher than 0.2 or lower than -0.2 and associated corrected P-value < 0.05). The 22 K NIAS gene expression data confirmed the correlation between *OsWRKY18 *and *OsWRKY4 *(see cluster A in Fig 2), and extended the clustering to the *OsWRKY22*, *OsWRKY100*, *OsWRKY53*, *OsWRKY78 *and *OsWRKY84 *genes (see Cluster I in Figure 3). These seven *OsWRKY *genes were found to be co-expressed in most conditions tested (e.g. flooding, drought and cold treatments) and in different plant organs (root, meristem, callus, panicle). This analysis revealed two additional clusters of co-expressed *OsWRKY *genes that were not identified by the OsWRKYARRAY analysis. Genes in cluster G, *OsWRKY24*, *OsWRKY8*, *OsWRKY42 *and *OsWRKY3 *are co-expressed in cold and drought conditions. Cluster H is constituted by the two genes *OsWRKY96 *and *OsWRKY50*, which have similar regulation profiles in flooding, cold and drought conditions.

Our findings are partially supported by previous comprehensive gene expression analysis of *OsWRKY *genes [23,40]. Ryu *et al*. [23], analysed the *OsWRKY *gene expression after infection with different pathogens (*Magnaporthe *strains and *Xanthomonas oryzae *pv *oryzae*) and treatment with hormone signalling molecules. Overall, between the two studies there is agreement for fifty percent of the genes identified as being differentially expressed upon *Magnaporthe *infection. It is important to stress that the cultivars (*indica *vs *japonica *varieties), pathogen strains, and plant-pathogen interactions (virulent/avirulent vs compatible/multi-avirulent/non host) used in the two studies were different, making difficult a direct comparison of the obtained gene expression results. The *WRKY *genes found to be induced only in one of the studies may reflect the existence of different responses to pathogen attacks and/or adaptation to different environmental conditions; these data may be pertinent to define the evolutionary history between different rice cultivars and their responses to the same pathogens. In a recent work [40], the *OsWRKY *gene family was analysed under different abiotic and phytohormone treatments and the authors showed that several *OsWRKY *genes were co-expressed at the tested conditions (cold, salt, drought, phytohormones). Interestingly, *OsWRKY4, OsWRKY43, OsWRKY61, OsWRKY53, OsWRKY63 *and *OsWRKY100* were found to be co-regulated upon different abiotic stress conditions, as well as in our experiments.

Comparing phylogenetic relationships and microarray-based gene expression clusters it was observed that the following pairs of closely related genes (*OsWRKY18 *and *OsWRKY4 *in cluster A, *OsWRKY71 *and *OsWRKY79 *in cluster E, *OsWRKY100 *and *OsWRKY53 *in cluster I) were co-expressed, reflecting recent duplications and potentially functional redundancy (see Figure 1). However, seven out of the nine identified clusters of co-expressed *OsWRKY*s contained sets of genes clearly belonging to different phylogenetic groups (see Figure 1). These findings suggest the existence of "complex networks" of *OsWRKY *genes contributing to orchestrate specific signal transduction pathways.

### Validation of OsWRKYARRAY by quantitative RT-PCR

To validate the results obtained with the OsWRKYARRAY, quantitative RT-PCR analysis (Q-PCR) of 58% (14 out of 24) of the differentially expressed rice *WRKY *genes was performed (13% of the whole gene family), to confirm their level of expression in leaves after *Magnaporthe *infection and osmotic stress treatment. The following fourteen genes were chosen for Q-PCR assays: *OsWRKY18, OsWRKY4, OsWRKY61, OsWRKY112, OsWRKY100, OsWRKY43, OsWRKY40, OsWRKY71, OsWRKY101, OsWRKY63, OsWRKY53, OsWRKY87, OsWRKY64 *and *OsWRKY115*. Quantitative expression of these genes was measured in samples obtained from new independent experiments carried out at the same conditions as were used to obtain RNA samples for the OsWRKYARRAY transcriptome analysis. RNA was extracted from leaves 24 hours after inoculation with the same three fungal strains that were used for the microarray experiments (*Magnaporthe *BR29, BR32 and FR13) and 5 hours post osmotic treatment, respectively. Results of the Q-PCR experiments from the four test conditions (three biological replicates/treatment) are reported in Table 2 and showed that eleven out of the fourteen tested genes (80%) were confirmed as differentially expressed with the associated P-value < 0.05. The Q-PCR data of three genes (*OsWRKY43, OsWRKY101 *and *OsWRKY115*) were not in agreement with those obtained in the microarray analysis. In conclusion, Q-PCR analyses confirmed the robustness of microarray results and validated our hypothesis of the existence of co-expressed cluster of *OsWRKY *genes. In particular, Q-PCR results confirmed that *OsWRKY4, OsWRKY18 and OsWRKY61 *(see cluster A in Figure 2) have very similar expression profiles, in agreement with the existence of OsWRKYs co-regulatory networks. Based upon these data, we decided to characterize in detail the occurrence of WRKY networks in the model plant *Arabidopsis thaliana*.

**Table 2:** Microarray validation by quantitative RT-PCR

**Treatment**	**microarray**	**QRT-PCR**			**Agreement**
***M. grisea *BR29**	Ma	Mq	st. dev.	P-val	

***OsWRKY4***	0.410	1.184	0.3818	0.0125	YES
***OsWRKY18***	0.629	2.0010	0.524	0.0119	YES
***OsWRKY43***	-0.324	-1.370	2.828	0.2403	NO
***OsWRKY61***	0.511	1.655	0.4857	0.0035	YES
***OsWRKY71***	1.053	1.7643	0.6223	0.0494	YES
***OsWRKY87***	NS	0.669	0.287	0.0098	qRT-PCR
***OsWRKY100***	-0.294	-0.081	0.825	0.9247	NO
***OsWRKY112***	0.407	1.829	1.245	0.0324	YES

***M. oryzae *BR32**	Ma	Mq	st. dev.	P-val	

***OsWRKY4***	0.444	0.653	0.1858	0.1011	NO
***OsWRKY18***	0.554	0.8351	0.453	0.0352	YES
***OsWRKY40***	0.481	0.875	0.6098	0.0478	YES
***OsWRKY43***	-0.505	-1.049	2.361	0.2616	NO
***OsWRKY61***	0.235	1.056	0.3291	0.0478	YES
***OsWRKY64***	-0.365	-1.006	0.715	0.0365	YES
***OsWRKY87***	0.325	0.480	0.185	0.0410	YES
***OsWRKY100***	-0.500	-0.838	0.256	0.0379	YES
***OsWRKY112***	0.427	1.508	0.755	0.0503	YES

***M. oryzae *FR13**	Ma	Mq	st. dev.	P-val	

***OsWRKY4***	0.557	0.985	0.4824	0.0229	YES
***OsWRKY53***	-0.673	-0.8908	0.1138	0.0209	YES
***OsWRKY61***	0.402	1.272	0.6398	0.0100	YES
***OsWRKY63***	0.539	2.169	0.379	0.0013	YES
***OsWRKY100***	-0.402	-1.631	0.402	0.0165	YES
***OsWRKY101***	0.506	0.992	0.698	0.0752	NO
***OsWRKY115***	0.670	0.8978	0.6437	0.069	NO

**OSMOTIC stress**	Ma	Mq	st. dev.	P-val	

***OsWRKY4***	-0.813	-1.290	0.745	0.0478	YES
***OsWRKY18***	-1.256	-1.400	0.595	0.0421	YES
***OsWRKY40***	-0.248	-1.355	0.757	0.0089	YES
***OsWRKY43***	0.270	0.293	0.457	0.3920	NO
***OsWRKY61***	-0.443	-1.570	0.349	0.0164	YES
***OsWRKY63***	-0.234	-0.658	0.017	0.2974	NO
***OsWRKY87***	-0.309	-1.320	0.272	0.0314	YES
***OsWRKY112***	-0.404	-0.848	0.368	0.0468	YES

Results of quantitative expression for the fourteen *OsWRKY *genes selected to validate the OsWRKYARRAY results are summarized. The expression level of each *OsWRKY *gene was measured in leaf samples infected with three *Magnaporthe *strains (BR29, BR32, FR13) and after osmotic stress treatment (see **Treatment **column). **Ma **is the log_2 _value of the ratio of expression levels between treated and control RNA samples obtained in the OsWRKYARRAY (see Table 1); **Mq **and **st.dev**. indicate log_2 _and standard deviation of ratio treated vs controls obtained by qRT-PCR, respectively. **P-val **indicates the P-value obtained with the statistical T-test. Results with an associated P-value > 0.05 were considered not significant and therefore are not reported. The "Agreement" column reports agreement among microarray and qRT-PCR results. **YES **with agreement in up/down regulation; **NO **without agreement in up/down regulation; qRT-PCR: indicates genes up/down regulated only in the qRT-PCR experiments. **NS **in the microarray colum indicates genes resulted not significant at the statistical analysis of the microarray data.

### WRKY co-regulatory networks

The integrated transcriptome results indicated that specific clusters of co-expressed rice *WRKY *genes are involved in response to a range of applied stress conditions. The clusters A, E and F comprised mostly *OsWRKY *genes belonging to the same phylogenetic groups and often closely related. These genes are likely to be derived from recent duplication events and, therefore, as it may be expected, to share similar expression profiles. On the other hand, the clusters B, C, D, G and H mainly consisted of members of distinct phylogenetic groups. The largest cluster (I) included both distantly-related and closely-related *OsWRKY *genes (see Figure 1).

To further investigate clusters of co-expressed *WRKY *genes in plants, data were collected from 2,000 Arabidopsis Affymetrix microarray experiments and correlation analysis based on the Pearson Correlation Coefficient (PCC) was carried out; scatterplots of individual gene pairs were obtained, as previously described by Toufighi *et al*. 2005 [41]. A scatter plot of the results obtained with two non-correlating (*AtWRKY35 *vs *AtWRKY40*) and two correlating (*AtWRKY33 *vs *AtWRKY40*) genes is presented in Additional file 4. The source and the processing of the gene expression data were described in detail in Menges *et al*. [42], but a new matrix was generated for this study. For every *AtWRKY *gene on the At Affymetrix microarray (61 out of the 74 *WRKY *genes present in the Arabidopsis genome), we calculated the untransformed PCC value (P-lin) with each of the other members of the gene family [see Additional file 5]. In the logarithm analysis (P-log), the gene expression data were transformed into logarithmic values before calculating the PCC [see Additional file 6]. We performed both P-lin analysis to pinpoint co-regulatory patterns occurring in only few microarray experiments (e.g. tissue- or condition-specific expression) and P-log analysis to better define the *WRKY *co-regulation in the presence of very different gene expression levels between the gene pairs under examination. To define the existence of *AtWRKYs *co-regulatory networks, values of Pearson Correlation Coefficient higher or equal to 0.6 were considered as significant both for the topology of the networks and for the number of represented genes. To validate the PCC threshold value used to obtain *AtWRKYs *co-regulatory networks, PCC analysis of the *AtMADS-BOX *gene family was also carried out. As for the topology, the appropriateness of using the 0.6 threshold value was confirmed by plotting the number of edges and the mean of edges/gene as a function of the threshold values [see Additional file 7]; this analysis clearly highlighted that, taking a threshold value of 0.7, the mean number of edges/gene significantly dropped by 30%, losing important information about the complexity of the network. In addition, by plotting the number of edges and number of genes as a function of the threshold values [see Additional file 8], it was observed that the number of genes is reduced by 30% in the P-lin and by 39% in the P-log analysis. Moreover, the number of edges dropped by 59% in the P-lin analysis and by 70% in the P-log analysis, when the threshold value was raised from 0.6 to 0.7. As a further supporting evidence the *AtMADS*-*BOX *PCC analysis was performed at 0.6 and 0.7 threshold value [see Additional file 9 and 10], as this gene family is experimentally well characterized at the molecular and genetic levels. This analysis revealed that the network of the *AtMADS*-*BOX *genes (involved in floral differentiation) is very robust, with 13 genes in the P-lin analysis with threshold value > 0.6 [see Additional file 9A] linked by 40 edges, 32 of which are backed up by molecular evidence for direct interaction (e.g. two hybrid, co-IP) or for involvement in ternary or quaternary complexes [43,44]. Moreover, 5 out of the 8 interactions lacking direct molecular evidence involved SEP2 which is an auto-activator in the two-hybrid assay and, therefore, could not be tested as bait. Two other separate networks of *MADS*-*BOX *genes were identified from our PCC analysis, which were backed up by molecular or genetic evidence: one network comprised *AGL18*, *-29*, *-30*, *-65*, *-66 *and *-104 *implicated in pollen maturation [45] and the second one *AGL67*, *-68*, *MAF4*, *MAF5 *and *FLF *involved in flowering transition [46]. The P-lin networks obtained with threshold value of 0.7 [see Additional file 9B] loses experimentally supported connections such as *AP1 *with *P1 *and *AP3*/*SP3 *with *SHP1*. In addition applying the threshold value of 0.7, *SEP4 *is absent in the main *AtMADS-BOX *network and the *MAF5 *gene is missing in the flowering one. The P-log analysis with threshold value of 0.6 [see Additional file 10A] keeps the same general topology as the P-lin one, albeit with a slightly reduced complexity (with 10 genes and 25 edges), whereas results obtained from the P-log analysis with threshold value of 0.7 [see Additional file 10B] reduces dramatically the number of genes and networks which are mostly experimentally validated. These data clearly showed that, carrying out the PCC analysis with a threshold value of 0.7, the number of genes represented in the network decreases significantly, and confirmed the appropriateness of using the 0.6 threshold value.

**Figure 4:**
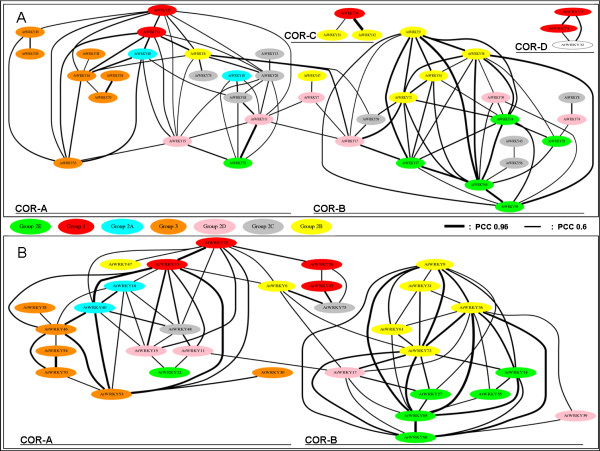
**Co-regulatory networks of Arabidopsis *WRKY *genes**. For each pair of *WRKY *genes, the Pearson Coefficient was calculated on untransformed values P-lin (see **Panel A**) and on log-transformed values P-log (see **Panel B**) to measure the correlation of expression levels, based on 2,000 Arabidopsis microarray experiments. Each pair of correlated *WRKY *genes (Pearson Correlation Coefficient value higher than 0.6) are shown in the figure with an edge connecting them. The thickness of the edges is proportional to the value of the Pearson Correlation Coefficient. Thick black line: Pearson Correlation Coefficient 0.96; Thin black line: Pearson Correlation Coefficient 0.6. Proximity of two genes on the graph is not indicative of their relatedness. The colours indicate different phylogenetic groups. See Additional files 5 and 6 for the specific numeric values of the P-lin and P-log correlation coefficient, respectively. The four identified co-regulatory neworks were indicated as: COR-A, COR-B, COR-C and COR-D.

P-lin analysis of *AtWRKY *genes revealed the existence of two major co-regulatory networks (COR-A and COR-B) and of two additional smaller networks COR-C and COR-D (Figure 4A). The P-log analysis confirmed the existence of the two interconnected COR-A and COR-B clusters (Figure 4B) while the other two smaller networks were not present. Taken together, the P-log and P-lin analyses revealed that more than 70% (45 out of 61) of the Arabidopsis *WRKY *genes analysed are co-regulated with other *WRKYs *[see Additional file 11]. The existence in COR-A of a sub-cluster constituted of *AtWRKY70, AtWRKY38, AtWRKY46 *and *AtWRKY54 *co-regulated genes in both P-lin and P-log analyses, was experimentally proven by Kalde *et al*. [22]. In the P-lin analysis the genes *AtWRKY70*, *AtWRKY38, AtWRKY46 *and *AtWRKY54 *are clustered together, whereas, *AtWRKY30 *and *AtWRKY55 *are apart, although they belong to the same group 3. This is in agreement with the results by Kalde *et al*. [22], who showed that the former four genes are induced by salicylate and pathogens, whereas the latter two are not differentially expressed at the same conditions. Moreover, the implication of the strongly co-regulated *AtWRKY25 *and *AtWRKY33 *genes (P-lin value 0.74) in the same signal transduction pathways and the functional redundancy of *AtWRKY11 *and *AtWRKY17 *(P-lin value 0.63) were reported by Andreasson *et al*. [47] and Journot-Catalino *et al*. [48], respectively. It is noteworthy to highlight that in the *AtWRKY *PCC analysis using a threshold value of 0.7 [see Additional file 12], the aforementioned subcluster of *AtWRKY70, AtWRKY38, AtWRKY46 *and *AtWRKY54 *genes and the connection between *AtWRKY11 *and *AtWRKY17 *were lost. As previously mentioned, P-lin analysis allowed us to highlight the existence of a correlation between two genes that are expressed in just a few conditions/treatments, while P-log highlights co-regulation between genes even in the presence of large differences in their expression levels. The small networks COR-C and COR-D were present only in the P-lin analysis (see Figure 4A), reflecting their co-expression only in few tested experimental conditions (data not shown). *AtWRKY3 *and *AtWRKY4 *genes (COR-D) were found to be specifically and rapidly induced upon infection with *Botrytis cinerea *and the virulent *P. syringae *pv. *tomato *strain DC3000 (*PstDC3000*). As a supporting evidence of their restricted but correlated role, the *wrky3wrky4 *double mutant plants exhibited more severe disease symptoms only following *Botrytis *infection [49]. On the other hand, the P-log analysis (Figure 4B) revealed the co-regulation (P-log value 0.73) of two (*AtWRKY18 *and *AtWRKY40*) of the three *WRKY *genes belonging to the group 2A, previously shown to physically interact [28].

In conclusion, our approach is validated by the presence in the COR-A and COR-B networks of recently duplicated genes tightly co-regulated not only *in silico *but also *in vivo*. Our work revealed that both COR-A and COR-B networks included significantly co-regulated *WRKY *genes belonging to distinct phylogenetic groups (see Figure 4 and Additional file 11); the *WRKY *PCC analysis is a valuable tool to unveil novel co-regulatory pathways between *WRKY *genes in plants.

### Integration of rice co-expression clusters and Arabidopsis WRKY co-regulatory networks

We attempted to link rice co-expression clustering data to Arabidopsis co-regulatory pathways and vice versa by search for respective orthologs. We used GreenPhylDB [50] to search for pairs of rice-Arabidopsis orthologs in the *WRKY *gene family with a bootstrap value greater than 50%. By this analysis we identified the following conserved *WRKY *networks between rice and Arabidopsis (see Table 3):

**Table 3:** Rice - Arabidopsis orthologs in *WRKY *co-regulatory pathways

**OsWRKY gene name**	**cluster Os-microarray**	**Phylogenetic Group in At-Os tree**	***At *putative ortholog**	**At CO-REG NETWORK**
***OsWRKY61***	**A**	1	*AtWRKY33*	Cor-A
***OsWRKY4***	**A/I**	2A	*AtWRKY18*	Cor-A
***OsWRKY18***	**A/I**	2A	*AtWRKY40*	Cor-A
***OsWRKY22***	**I**	1	*AtWRKY22*	Cor-A
***OsWRKY53***	**F/I**	3B	*AtWRKY46 or AtWRKY53*	Cor-A
***OsWRKY78***	**I**	2C	*AtWRKY75*	Cor-A
***OsWRKY100***	**C/I**	3B	*AtWRKY46 or AtWRKY53*	Cor-A
***OsWRKY84***	**I**	2E	*AtWRKY69*	Cor-B
***OsWRKY43***	**C**	2B	*AtWRKY6 or AtWRKY31*	Cor-A/B
***OsWRKY71***	**E**	3B	*AtWRKY46*	Cor-A
***OsWRKY79***	**E**	3B	*AtWRKY54 or AtWRKY70*	Cor-A
***OsWRKY19***	**B**	3B	*AtWRKY54 or AtWRKY70*	Cor-A
***OsWRKY37***	**B**	1	*AtWRKY26 or AtWRKY2*	-
***OsWRKY112***	**B**	1	*AtWRKY34*	Cor-C
***OsWRKY24***	**G**	2C	*AtWRKY8*	Cor-B/Cor-A
***OsWRKY3***	**G**	1	no ortholog	-
***OsWRKY8***	**G**	3C	no ortholog	-
***OsWRKY42***	**G**	2D	*AtWRKY11*	Cor-A
***OsWRKY11***	**F**	3C	no ortholog	-
***OsWRKY38***	**F**	3C	no ortholog	-
***OsWRKY40***	**D**	3C	no ortholog	-
***OsWRKY48***	**D**	1	no ortholog	-
***OsWRKY86***	**D**	2B	*AtWRKY31*	Cor-B

*OsWRKY *genes belonging to each of the nine identified co-expression clusters in rice are listed together with their assigned phylogenetic group, according to the At-Os phylogenetic analysis [see Additional file 2]. When identified, the putative Arabidopsis ortholog/s with a bootstrap value higher than 50% is/are reported along with its/their affiliation to a co-regulatory network, as obtained by P-lin correlation analysis (Figure 4). **no ortholog **indicates the absence of identified corresponding Arabidopsis gene.

- the Arabidopsis orthologs (*AtWRKY18, AtWRKY40 *and *AtWRKY33*) of the three rice genes of cluster A in the OsWRKYARRAY (*OsWRKY61*, *OsWRKY4 *and *OsWRKY18*) were significantly connected in the COR-A network. In particular, the two orthologs, *AtWRKY18 *and *AtWRKY40*, were connected in P-log analysis and, as aforementioned, it was reported that they physically interact *in vitro *and *in vivo *[28].

- the Arabidopsis orthologs (*AtWRKY46, AtWRKY70/AtWRKY54*) of the two rice genes of cluster E (*OsWRKY71 *and *OsWRKY79*) were found to be connected within the COR-A network; these findings were experimentally supported by their very similar profiles of gene expression upon pathogen attack [22].

- the six genes belonging to the rice co-expression cluster I (*OsWRKY4,-18,-22,-53,-78,-100*) had all Arabidopsis orthologs belonging to COR-A; five of these identified *AtWRKY *orthologs were directly pairwise connected within the COR-A network.

As regards to the remaining rice *WRKY *genes shown to be part of co-expression clusters, in most cases, it was not possible to find their respective orthologs in Arabidopsis. In particular, four of them belong to the OsWRKY 3C group, which has not orthologs in Arabidopsis [see Additional file 2]. The role of these *WRKY *genes and of the 3C rice-specific phylogenetic group in defence related signalling pathways deserves further investigations at the functional level.

## Discussion

### Rice co-expression WRKY clusters

In the present work we carried out whole *OsWRKY *gene family transcriptome analysis upon *Magnaporthe *infection and osmotic stress treatment to identify clusters of genes differentially co-expressed upon biotic and abiotic stress conditions.

The genes *OsWRKY6, -18, -61, 71*, found to be differentially expressed in the OsWRKYARRAY experiments, were previously described as being involved in biotic and abiotic stress responses [51-54]. In particular, *OsWRKY18*, which resulted to be significantly regulated in our biotic/abiotic stress experiments, was reported as being modulated in roots during microbial colonization [55], after application of salicylic acid, methyl jasmonate, 1-aminocyclo-propane-1-carboxylic acid and in response to both wounding and pathogen infection [52].

To extend our transcriptome analysis we took advantage of a large set of gene expression data extrapolated from the 22 K NIAS array experiments. By integrating the data from both microarrays, nine co-expression *WRKY *gene clusters were identified (see Figures 2 and 3), some of them restricted to specific experimental conditions, while others found in a larger set of them. Despite the relatively low ratios of the expression levels in both rice microarray experiments of the differentially-regulated *WRKY *genes, the majority of them were confirmed by qRT-PCR analyses. In fact, most plant transcriptional factors are tightly and, often, only transiently up- or down-regulated upon stress stimuli making it difficult to capture their peak of induction/repression [56-58]. Moreover, in the case of infection with fungal pathogens, as *Magnaporthe*, only a few cell layers (e.g. epidermal cells) are implicated in the plant responses [38], while gene expression studies rely on bulked leaf material, causing a "dilution" effect of the detected gene expression levels [59,60]. In a number of cases only *in situ *hybridisation or laser capture microdissection (LCM) coupled with microarray experiments revealed the exact gene expression patterns of specific members in transcriptional factors gene families (e.g. *MADS-BOX *genes in ovules, meristem) [61,62]. Recently, LCM technology was also successfully applied to the study of plant-microbe interactions [63].

The integration of the gene expression clusters to the phylogenetic data of the whole *OsWRKY *gene family (see Figure 1) highlighted that also genes belonging to clearly distinct clades were significantly co-expressed. Closely-related *OsWRKY *genes, likely to be derived from recent duplication events, were found to be tightly co-regulated; this was expected, since it was shown that recently duplicated *WRKY *genes often keep the same role in signal transduction, representing a typical case of functional redundancy [37]. On the other hand, the *OsWRKY *CORs would indicate the existence of WRKY protein complexes where WRKYs belonging to different subgroups bind to different cis-regulatory elements, thus giving them different targets that are expressed under the same conditions, tissue and timing. The identification of co-regulated *WRKY *genes would allow researchers making more-informative choices of double and triple mutant combinations to circumvent redundancy or test for potential protein-protein interactions to functionally investigate the relevance of specific WRKY complexes in pathogen resistance, tolerance to abiotic stress, hormonal balance and plant development.

### At WRKY co-regulatory networks

To address the existence of co-regulatory pathways of phylogenetically unrelated *WRKY *genes in plants we carried out Pearson Correlation Coefficient (PCC) analysis of Arabidopsis Affymetrix transcriptome data from 2,000 experiments. All Affymetrix array experiments were carried out by the same laboratory (NASC) with standardized normalization process thus facilitating the creation of robust and reliable PCC matrices. The generated matrices for *AtWRKY *genes using both untransformed [see Additional file 5] and logarithm-transformed [see Additional file 6] expression data were analysed at different threshold values of PCC. The appropriateness of the applied threshold value of 0.6 was validated using the set of available data for the *MADS-BOX *gene family of transcription factors [see Additional file 7, 8]. We took advantage of the in-depth knowledge of the *MADS-BOX *signal transduction pathways and protein-protein interactions to validate the factual existence of the correlations identified in our PCC approach. In most cases the identified co-regulatory edges well reflected the existing genetic evidences described in the literature. Moreover, most of the PCC-identified networks supports specific sets of *MADS-BOX *genes in the same signal transduction pathways and their tight co-expression in specific cell layers [64,65].

The PCC analysis (see Figure 4) highlighted the presence of two main interconnected co-regulatory networks of phylogenetically distinct *AtWRKY *genes (COR-A, COR-B). Such networks represent powerful tools to identify candidate partners of *WRKY *genes of interest or to investigate experimentally the existence of interactions between WRKY proteins *in vivo *(e.g. co-immunoprecipitation analysis). The presence in COR-A of *AtWRKY18*, *AtWRKY40*, *-38*, *-46*, *-54*, *-70, 33 *and *-25 *showed that the PCC approach was able to identify *WRKY *genes previously described as being part of a functional network involved in response to stress stimuli. In fact, *AtWRKY18 *and *AtWRKY40 *are pathogen-induced genes and known to physically interact [28]. *AtWRKY38*, *AtWRKY46*, *AtWRKY54 *and *AtWRKY70 *are also pathogen-induced genes and three of them are known to act in the same regulatory pathway. Moreover, the application of salicylic acid to *wrky54 *mutants altered the expression patterns of *AtWRKY38 *and *AtWRKY70 *genes [22]. The COR-A network also included *AtWRKY *genes found to be involved in Systemic Acquired Resistance (SAR) by Wang *et al*. [29], in a study aimed to identify targets of *NPR1*, an essential regulator of plant SAR. Among these targets, the authors found eight AtWRKYs of which five were shown to be part of the complex transcriptional regulatory network of SAR. In our PCC analysis of *AtWRKY *networks, four of these five genes (*AtWRKY18*, -*54*, -*53 *and -*70*) not only belonged to the same group (COR-A), but they were also tightly correlated (Fig 4A). In this study, the authors stated that, in addition to *AtWRKY46*, *AtWRKY53*, *AtWRKY54*, a related gene of *AtWRKY70 *is required to fully silence salicilate biosynthesis, yet to be identified. In both the P-lin and P-log analysis *AtWRKY38 *is significantly connected to these co-regulated genes and, therefore, it may be the best candidate to be functionally characterized.

Most of the previously-mentioned genes were studied as they are phylogenetically related [22,28]. However, our PCC analysis of the *AtWRKY *genes suggested that the interaction in co-regulatory networks may occur also between phylogenetically unrelated *WRKY *genes, such as *AtWRKY40*, *AtWRKY6, AtWRKY33 *and *AtWRKY46 *belonging to different groups (2A, 2B, 1 and 3, respectively).

The topology of the COR-A network predicted the presence of further genes that could be involved in the same signal transduction pathway. A role for these genes in plant defence has yet to be defined, but according to the Pearson co-regulatory analysis they are interesting candidates to be tested at the genetic and biochemical level. Similarly, the co-regulatory network Cor-B indicated that several unrelated *WRKY *genes, functionally uncharacterized to date, are likely to interact among each other. Genetic analysis of those genes as part of a regulatory network rather than as single genes may give further insights into their function and role in specific signalling pathways. In COR-C the *AtWRKY34 *and *AtWRKY42 *genes were found to be strongly connected by a PCC value of 0.9; these two genes, together with *AtWRKY31 *were only seen in the P-lin analysis as their expression was found to be restricted only to a very specific plant tissue (data not shown). The COR-D network, only present in the P-lin analysis, was constituted of the three genes *AtWRKY3*, *AtWRKY4 *and *AtWRKY32*. While the genetic interaction between *AtWRKY3 *and *AtWRKY4 *both belonging to the phylogenetic group 1 was previously demonstrated [49], the association of the yet unassigned *AtWRKY32 *to the other two genes and its potential role in WRKY-mediated pathogen signal transduction pathways (e.g. resistance mechanisms to *B. cinerea*) remains to be elucidated. Our PCC analysis enables scientists to formulate new working hypotheses involving *AtWRKY *genes belonging to distinct phylogenetic groups to dissect specific regulatory pathways in plants.

### Orthology rice - Arabidopsis

A large set of microarray data is vital to build up detailed and reliable co-regulatory networks. Among plants, this can be achieved, to date, only for *Arabidopsis thaliana *due to the large and consistent expression dataset. However, the Arabidopsis co-regulatory networks can be used as reference for other species where a smaller set of expression experiments is available. In addition, when information of orthology is available between Arabidopsis and another species, this approach can identify the existence of genes involved in a common biological process or extend their number, even if expression data are not sufficient to reveal the existence of co-regulatory networks [25]. This approach was proven to be successful and highly informative for *OsWRKY *genes in this study. In fact, we found 20 pairs of orthologous genes among rice and Arabidopsis and 8 of them were co-regulated in both species, integrating our microarray, Q-PCR and PCC results.

In summary, our first attempt to correlate specific *OsWRKY *co-expression clusters to *AtWRKY*-COR groups revealed the existence of one large (cluster I in rice) and two smaller (cluster A and E in rice) conserved co-regulatory networks between the two model plants. This will now open the route to test the functional conservation of the identified clusters of *WRKY *genes between the two species and their involvement in the same signal transduction pathways. However, the difficulty in finding orthologs between rice and Arabidopsis *WRKY *genes, due to successive rounds of duplications in both species and to the existence of Monocot and Dicot-specific phylogenetic clades, calls for the need to develop suitable transcriptome resources to carry out PCC analysis in rice. Only this systematic analysis will enable researchers to develop working hypotheses on co-regulatory signal transduction pathways of rice *WRKY *genes to be experimentally tested.

## Conclusion

Ülker and Somssich [6] pointed out that to assign a specific function to members of this complex gene family "of imminent importance is to uncover WRKY-interacting proteins that assist in regulating the transcription of genes". Here we proposed a validated and innovative approach that aims at finding such interacting proteins relying not on their sequence similarity, but rather on co-regulation at the transcriptome level. Our integrated rice-Arabidopsis co-expression approach showed the existence of large co-regulatory networks of *WRKY *genes in plants. The PCC analysis revealed that closely-related *AtWRKY *genes, known to be involved in the same signal transduction pathways (and often functionally redundant), are also strongly co-regulated with other phylogenetically distantly-related members of the *WRKY *gene family. The in-depth analysis of *WRKY *COR networks will surely contribute to unveil the function of *WRKY *genes, as a more targeted genetic analysis will be possible on sets of candidate genes, shown to be significantly co-regulated. Moreover, the existence of complex regulatory pathways clearly supports the existence of cascades of WRKY signal transduction steps, as shown for *MADS-BOX *and MAP kinase genes [66], yet to be defined.

In conclusion our rice-Arabidopsis integrated approach strongly supports the existence of cross-regulatory pathways by *WRKY *genes possibly via specific feedback mechanisms, as recently highlighted by Pandey *et al*. [25].

The PCC analysis presented in this manuscript represents a powerful tool applicable to gene families of other classes of transcriptional factors, contributing to define regulatory networks in plants activated in response to biotic and abiotic stress stimuli.

## Methods

### WRKY gene sequences

Nucleotide sequences to design oligos have been retrieved searching for PFAM ID PF03106 as query in the Rice Annotation Release 5 at TIGR . The PFAM PF03106 is the name of the WRKY domain stored in the PFAM database, a large collection of protein domain families, which provides multiple sequence alignments and Hidden Markov Models (HMMs). When more than one alternative splicing sequence was found for the same locus, only the longest one was used. Further sequences previously found as *WRKY *gene in previous releases of TIGR but not confirmed in the last one were kept anyway. A search for *WRKY *genes not annotated as WRKY by TIGR was perform with tblastn on Genbank using the following consensus sequence: KPRFAFMTKSEVDILDDGYRWRKYGQKMIKNNPYPRSYYRCTMAKGCVKKQVERCSDDPIIVITTYEGQHNHPWP as a query filtering for E value < 10^-13^. A summary table specifying nomenclature used in this article of the retrieved 104 *OsWRKY*, gene locus in TIGR nomenclature and names provided in earlier publications [17,34] are reported in Additional file 1. For each gene, a gene-specific sequence of 60 nucleotides was designed generally in the 3' end of the gene and, in any case, out of the conserved domain. Four replicates of each oligo were spotted on slides together with oligos for positive, negative, and 7 housekeeping genes. A complete list of spotted genes and their sequences is provided in Additional file 3 and submitted on-line at GEO  with this accession number: GSE5819.

### Phylogenetic analyses

We based our evolutionary reconstruction of the *WRKY *family on the multiple alignment of the amino acidic sequence of the WRKY domains. We made two major reconstructions: one with all the rice members of the *WRKY *gene family and the second with all the WRKY domains of rice and Arabidopsis. In the latter, the non-plant sequences of *Giardia lamblia*, *Dictyostelium discoideum *and *Chlamydomonas reinhadrtii *were also included. The final set of proteins was composed of 122 sequences for the OsWRKY tree and of 204 for the Arabidopsis-Os WRKY tree. We built up a multiple sequence alignment (MSA) using MUSCLE [67] with the maximum number of iterations set to 1000. We derived Maximum Likelihood (ML) phylogenetic inferences using PHYML [68], applying the JTT matrix. Our model of sequence evolution assumed that there were two classes of sites, one class being invariable and the other class being free to change. The rate variation across these sites was assumed to follow a gamma shape distribution calculated using a discrete approximation with eight categories of sites. One hundred bootstrap replicates were used to support the hypotheses of relationships. The tree image was produced using iTOL [69].

### Interaction with Magnaporthe grisea

Rice plants (*Oryza sativa *L. ssp japonica) cv. Nipponbare were grown from seeds in a greenhouse in trays of 40 × 29 × 7 cm filled with compost (7/8 Neuhaus compost N 9, 1/8 pouzzolane) under a 27°/22°C day/night temperature, 60% humidity. Twenty seeds were sown in rows with 6 rows per genotype. Nitrogen fertilization with 8.6 g of nitrogen equivalent was done at 7 and 2 days before inoculation. The same conditions were applied to control (mock) and infected plants.

### Magnaporthe grisea isolate cultures

Three fungal isolates with different geographic origins were chosen from the *Magnaporthe *(Hebert) Barr strain collection (CIRAD, Montpellier). *Magnaporthe oryzae *FR13 is a rice isolate, *Magnaporthe oryzae *BR32 is a non-rice isolate from wheat and *Magnaporthe grisea *BR29 was isolated from crabgrass. These strains were cultured in Petri dishes containing 20 mL of medium composed with 20 g × L^-1 ^rice seed flour, 2.5 g × L^-1 ^yeast extract, 1.5% agar (Merck). After autoclaving, 500,000 units of Penicillin G (Sigma) were added aseptically by filter sterilizing. The cultures were then placed in a growth chamber with a 12 h photoperiod and a constant temperature of 25°C for 7 to 9 days prior to inoculation.

### Inoculation

Conidia were harvested from plates by rinsing with sterile distilled water and filtering through two layers of gauze. Inoculations with *Magnaporthe *strains were performed with rice plantlets 2 weeks after sowing by spraying with conidial suspensions. Thirty mL of either a 100,000 (for FR13) or 300,000 (for BR29 or BR32) conidia × mL^-1 ^suspension with 0.5% gelatin were sprayed on each tray (60 plants). Control plants were sprayed with a solution of water with 0.5% gelatin (mock-treated leaves). Treated and control rice plants were then kept together for 16 hours in a controlled climatic chamber at 25°C and 95% relative humidity. Leaf tissue was then harvested 24 hours after inoculation for total RNA extraction. To obtain RNA for the microarray experiments, each treatment was repeated twice on independent assays. Additional independent experiments were carried out in three biological replicates to obtain RNA for the Q-PCR and validate OsWRKYARRAY results.

### Abiotic stress

Dehisced seeds from Nipponbare genotype were sterilized with 15% (v/v) sodium hypochlorite for 30 minutes and then rinsed with distilled water. Seeds were germinated in Petri dishes at 28°C on Whatman paper soaked in deionized water. After 4 days, rice seedlings were transferred in pots containing pouzzolane and allowed to growth under controlled conditions with a 28/25°C day/night temperatures, 12 hours photoperiod and 55-65% humidity for 2 weeks in Yoshida modified solution (Yoshida, 1981): 0.7 mM KNO_3_, 1.2 mM Ca(NO_3_)_2_, 1.6 mM MgSO_4_, 0.5 mM (NH_4_)_2_SO_4_, 0.8 mM KH_2_PO_4_, 60 μM FeEDTA, 20 μM MnSO_4_, 0.32 μM(NH_4_)_6_Mo_7_O_24_, 1.4 μM ZnSO_4_, 1.6 μM CuSO_4_, 45.2 μM H_3_BO_3_. Medium pH was adjusted to 5.0 twice a day. Seedlings at the 5 leaves stage without any visible tiller were carefully selected for stress treatments. For osmotic stress, 100 mM of mannitol was added in the hydroponic solution, whereas in the control (non-stressed) mannitol was not added to the solution. Root and leaf tissues were harvested 1 hour and 5 hours after the beginning of stress treatment. The harvested tissues were immediately frozen in liquid nitrogen and stored at -80°C. Tissues of control plants were collected at the same conditions and time as stressed plants. Each treatment was repeated twice, on independent assays for the microarray experiments. Additional independent experiments were carried out in two biological replicates, to obtain RNA for the qRT-PCR and validate OsWRKYARRAY results. Leaf samples from treated and control plants were harvested 5 hours after mannitol application.

### RNA extraction

Each biological replicate was obtained by pooling three leaves (third leaf) harvested from three different plants (mock/infected) for each treatment. Total RNA of each biological replicate was purified, using the TRIZOL protocol (Invitrogen, Carlsbad, CA), following the manufacturer's instructions. Total RNA was quantified using a NanoDrop ND-1000 Spectrophotometer; RNA with an absorbance A260/A280 ratio > 2.0 was tested for quality and integrity using the Agilent 2100 Bioanalyzer (Agilent).

### Microarray hybridisation

The customized OsWRKYARRAY microarrays were produced using a Virtek ChipWriter Pro contact printing robot. Hundred thirty oligos of 60-mers representing rice *WRKY *genes and positive and negative controls were printed onto Corning GAPSII (gamma amino propyl silane) slides at a final concentration of 20 μM. Ten μg of total RNAs were used for Cy3 or Cy5 labelling using the Cyscribe First-Strand cDNA labelling kit (Amersham). The labelled cDNA samples were purified using the CyScribe GFX Purification Kit (Amersham) and concentrated using a microcon YM-30 filter (Millipore). Five ng of Luciferase RNA (Promega) was added to each RNA sample prior labelling and used as spiking control. For array hybridisation, Cy3 and Cy5 labelled cDNA probes were mixed with Calf thymus DNA (Sigma) and EGT hybridisation buffer (Eurogentec) and hybridized to the microarray (Eurogentec) at 42°C in a humid chamber (Corning) for 16 hours. Arrays were washed 5 minutes in a 0.2×SSC-0.1% sodium dodecyl sulfate solution then 5 minutes in 0.2×SSC. The arrays were spin-dried and scanned using the Axon 4100A Scanner. The hybridisation data were collected using the GenePix Pro 3.0 software. Dye swaps for each experiment were performed and hybridisations repeated twice for experiments BR29, BR32, osmotic stress (leaves) at 5 hours, and for osmotic stress (roots) at 5 hours; only once for FR13 and osmotic stress (roots) 1 hour. Two biological replicates have been tested for each analysed condition. Three negative (Drosophila melanogaster lysozyme C, Drosophila melanogaster myosin 61F, Drosophila melanogaster Male-specific RNA 57Db), and four positive control (PR1 and PBZ1, dehydration-stress inducible protein and no apical meristem) were included. Housekeeping were the following genes: actin, zinc finger, cathepsin b-like cysteine proteinase, polyubiquitin, glyceralde-3-phosphate dehydrogenase, vacuolar proton-translocating ATP-ase subunit [see Additional file 3].

### Microarray and clustering analysis

*OsWRKY *expression data were extracted from results of 30 hybridisation experiments with the 22 K NIAS array  of which 17 were involving abiotic stress treatments; each value represents the mean of three independent hybridisation experiments. Both OsWRKYARRAY and NIAS 22 K microarray data were normalized using Limma [70] a package of the statistical software R, part of Bioconductor . Normalization on total signal was performed using the "loess function", but giving a differential weight (10 times higher) to housekeeping genes and DNA. A linear model was then applied to test the null hypothesis that the log of the ratio treatment/control was equal to 0. Associated P-values were corrected for false discovery rate [71]. For presence-absence of transcript, for every slide expression level (log of the average of two channels) of replicates of every gene was analysed with statistical T-test, if significantly different from expression level of negative control (Mst_57_Db, Myo61 and LysC). Genes which passed the test with a P-value < 0.001 were considered expressed. Raw data can be found at GEO (NCBI) with accession numbers GSE5819 (WRKYARRAY), GSE7531 and GSE7532 (NIAS). Clustering of rice data was performed using EPICLUST, a module of Expression Profiler . Default parameters were applied and a hierarchical clustering analysis was carried out using linear correlation based distance (Pearson) to calculate similarity matrix and UPGMA. Image of P-values were obtained using R on a computer with Ubuntu Linux installed.

### Real-time quantitative RT-PCR

Leaf samples were obtained in new independent experiments carried out specifically to biologically validate OsWRKYARRAY results. The RNA (800 ng) was treated with the DNase I (Fermentas), and 500 ng of the treated RNA was reverse-transcribed with High Capacity cDNA Reverse Transcription Kit (Applied Biosystem) using an oligo(dT) primer following manufacturer's recommendations. The cDNA synthesis reactions were treated with RNAse H, diluted hundred fold in sterile water, and 2,5 μl of the diluted cDNA served as template for PCR. For quantitative PCR, 2× Power SYBR Green PCR Master Mix (Applied Biosystem) were used according to manufacturer's recommendations on a 7900HT Fast Real-Time PCR System using version SDS 2.2.2 software (Applied Biosystems) to analyse raw data. Specific primer pairs were designed for 14 *WRKY *full-length cDNAs using Primer3 software and ordered from Sigma-Aldrich Company Ltd. (Haverhill, UK). Primer specificity was assessed by sequencing PCR products. Primer sequences are shown in Additional file 13. The expression level of each gene was measured in leaf samples infected with the three *Magnaporthe *strains and after osmotic stress treatment (4 different conditions). Results with an associated P-value > 0.05 were considered not significant and therefore are not reported in Table 2. The PCR was carried out in a total volume of 10 μL containing 0.3 μM of each primer, 1× Power SYBR Green PCR Master Mix (Applied Biosystems). Reactions were amplified as follows: 95°C for 10 min, then 40 cycles of 95°C for 15 sec, 60°C for 1 min. The absence of genomic DNA and non-specific by-products of the PCR amplification was confirmed by analysis of dissociation curves. For each primer pair, appropriate calibration curves were first obtained with different dilutions (0.1, 0.04, 0.02, 0.01, 0.004, 0.002, 0.0010) and were accepted when the correlation coefficient was ≥ 0.99 and the efficiency between 95 and 105%. All calculations for relative quantification were performed as described in Pfaffl [72] using a mathematical model to determine the relative quantification of the target gene compared with the reference gene (actin) from an inoculated plant versus a control (mock) one. Statistical significance of the difference between mock and infected was assessed by T-test analysis.

### Pearson correlation

Pearson correlation values were calculated essentially as described by Toufighi *et al*. [41] for the 'Expression Angler'. To this purpose a Visual C++ based program was developed (P. Morandini, L. Mizzi, unpublished) to calculate the correlation value from the data obtained with the ATH1 GeneChip from Affymetrix and deposited at the NASC array database  as of September 2008. For the calculation of Pearson coefficient from log values, data were simply transformed into log before calculating the correlation value. From such values, networks of Arabidopsis *WRKY *genes were produced using the program dot . The input text file for dot was prepared using a script that filtered *WRKY *genes with a reciprocal coefficient of 0.6 or higher from the complete table of Pearson coefficient. Intensity of arrow colours are proportional to the coefficient between each pair of *WRKY *genes. A more detailed explanation of the method used is reported in Menges *et al*. [42] in the section *Global expression correlation analysis *in *Methods*.

## Authors' contributions

SB and PA drafted the manuscript. SB performed the sequence search, carried out the gene family annotation, analysed OsWRKYARRAY expression data with statistical analysis, performed gene clustering. PA performed the stress experiments to validate OsWRKYARRAY, carried out quantitative Q-PCR and relevant statistical analysis. OFR and AB performed the stress experiments, provided the RNA for microarray hybridisation. OFR critically revised the manuscript. IF carried out the phylogenetic and orthology analyses, producing OsWRKY and Os-At WRKY tree figures. LM carried out PCC analyses and edited co-regulatory networks for *AtWRKY *and *MADS-BOX *genes. KS contributed to gene expression analysis using 22 K rice oligo microarray system. SK critically revised the manuscript. PM calculated Pearson coefficient from Arabidopsis microarray data, analysed and described co-regulatory networks and critically revised the manuscript. MEP conceived the initial research project. PP supervised and coordinated all experimental and analytical activities that led to the present publication and curated the manuscript preparation and revision. All authors read and approved the final manuscript.

## Supplementary Material

Additional file 1 ***OsWRKY *gene list**. List of the retrieved 104 *OsWRKY *genes with their corresponding gene names used in this article together with their ID used for the OsWRKYARRAY microarray analysis. Gene locus in TIGR nomenclature (release 5) and names provided in earlier publications are reported. Click here for file

Additional file 2 **Arabidopsis - rice WRKY phylogenetic tree**. Phylogenetic tree of rice and Arabidopsis WRKY domains obtained with the Maximum Likelihood method using PHYML [68]. Both the N and the C WRKY domains were considered for those proteins bearing two domains. Bootstrap values higher than 50 are indicated on the nodes. The sequences of *Giardia lamblia*, *Dictyostelium discoideum *and *Chlamydomonas reinhadrtii *were included. The tree image was produced using iTOL software [69]. The three distinct sub-groups of group 3 identified in this study are indicated as 3A, 3B and 3C. Click here for file

Additional file 3 ***OsWRKY *microarray oligonucleotides**. List of the set of oligonucleotides used for the OsWRKYARRAY microarray together with their ID and sequences. Click here for file

Additional file 4 ***AtWRKY genes scatter plots***. Typical scatter plot of the expression level of two pairs of *AtWRKY *genes across the set of Arabidopsis microarray experiments used for the Pearson Correlation Coefficient analysis. Each grey dot represents the simultaneous expression level of the two genes in one microarray experiment. **A**: The expression level of *AtWRKY40 *is not correlated with the expression of *AtWRKY35*. **B**: A strong correlation is present between *AtWRKY40 *and *AtWRKY33*. Click here for file

Additional file 5 **Linear Pearson Correlation Matrix of *AtWRKY *genes**. Correlation matrix of the untransformed Pearson Correlation Coefficient (PCC) values of the set of *AtWRKY *genes under examination. Click here for file

Additional file 6 **Logarithmic Pearson Correlation Matrix of *AtWRKY *genes**. Correlation matrix of the logarithmic-transformed Pearson Correlation Coefficient (PCC) values of the set of *AtWRKY *genes under examination. Click here for file

Additional file 7 **Plot of edges and mean of edges/gene vs PCC threshold value**. Plots of the number of edges (Y axis on the left) and mean of edges/gene (Y axis on the right) as a function of the PCC threshold values in the linear Pearson Correlation Coefficient analysis (P-lin) of the Arabidopsis *MADS-BOX *(above) and *WRKY *(below) genes. Click here for file

Additional file 8 **Plot of edges and number of genes vs PCC threshold value**. Plots of the number of edges and number of genes as a function of the PCC threshold values in the P-lin linear (above) and log-transformed (below) Pearson Correlation Coefficient analysis of the Arabidopsis *WRKY *(*AtWRKY*) genes. Click here for file

Additional file 9 **P-lin co-regulatory networks of Arabidopsis *MADS-BOX *genes**. Co-regulatory networks of Arabidopsis *MADS-BOX *genes obtained using untransformed Pearson Correlation Coefficient analysis (P-lin analysis). **A**: PCC threshold value of 0.6 **B**: PCC threshold value of 0.7. Unbroken lines indicate experimentally validated edges reported in literature; broken lines indicate edges not yet experimentally validated. The thickness of the edges is proportional to the value of the Pearson Coefficient. Thick black line: Pearson Correlation Coefficient 0.96; Thin Black Line: Pearson Correlation Coefficient 0.6 (in panel A) and 0.7 (in Panel B); the proximity of two genes on the graph is not indicative of their relatedness. Click here for file

Additional file 10 **P-log co-regulatory networks of Arabidopsis *MADS-BOX *genes**. Co-regulatory networks of Arabidopsis *MADS-BOX *genes obtained using log-transformed Pearson Correlation Coefficient analysis (P-log analysis). **A**: PCC threshold value of 0.6 **B**: PCC threshold value of 0.7. Unbroken lines indicate experimentally validated edges reported in literature; broken lines indicate edges not yet experimentally validated. The thickness of the edges is proportional to the value of the Pearson Coefficient. Thick black line: Pearson Correlation Coefficient 0.96; Thin Black Line: Pearson Correlation Coefficient 0.6 (in panel A) and 0.7 (in Panel B); the proximity of two genes on the graph is not indicative of their relatedness. Click here for file

Additional file 11 **List of Arabidopsis *WRKY *genes present in the co-regulatory**. List of the Arabidopsis *WRKY *genes with their affiliation to the different co-regulatory networks according to the P-lin and P-log Correlation analysis (see Figure 4) and their assigned phylogenetic group (see Additional file 2). Click here for file

Additional file 12 **Co-regulatory networks of Arabidopsis *WRKY *genes**. Co-regulatory networks of Arabidopsis *WRKY *genes obtained with the PCC threshold value of 0.7 in the untransformed (P-lin) (panel **A**) and log transformed (P-log) Pearson Correlation Coefficient analysis (panel **B**). The thickness of the edges is proportional to the value of the Pearson Correlation Coefficient. Thick black line: Pearson Correlation Coefficient 0.96; Thin Black Line: Pearson Correlation Coefficient 0.7. The proximity of two genes on the graph is not indicative of their relatedness. Click here for file

Additional file 13 ***OsWRKY *primer sequences used for quantitative RT-PCR analysis**. The sequences of primers used to analyse the expression levels of 14 *OsWRKY *genes by quantitative RT-PCR analysis are listed. Click here for file
