# Supplementary figures and images for: Characterization of WRKY co-regulatory networks in rice and Arabidopsis
Source: BMC Plant Biol. 2009 Sep 22;9:120. doi: 10.1186/1471-2229-9-120 (PMC2761919; doi:10.1186/1471-2229-9-120)

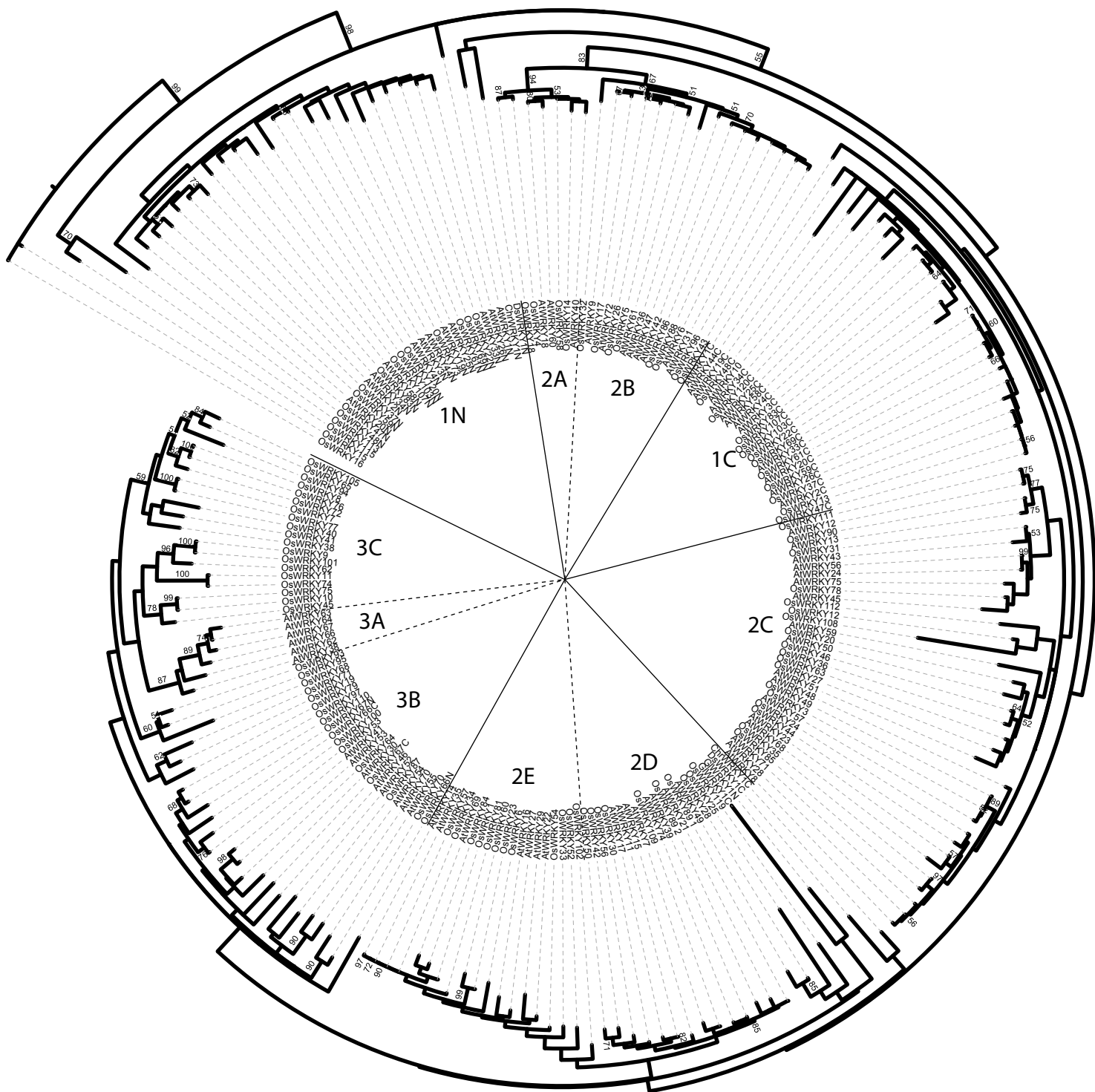

Supplement: Additional file 2 — Arabidopsis - rice WRKY phylogenetic tree. Phylogenetic tree of rice and Arabidopsis WRKY domains obtained with the Maximum Likelihood method using PHYML [68]. Both the N and the C WRKY domains were considered for those proteins bearing two domains. Bootstrap values higher than 50 are indicated on the nodes. The sequences of Giardia lamblia, Dictyostelium discoideum and Chlamydomonas reinhadrtii were included. The tree image was produced using iTOL software [69]. The three distinct sub-groups of group 3 identified in this study are indicated as 3A, 3B and 3C. [file 1471-2229-9-120-S2.pdf]

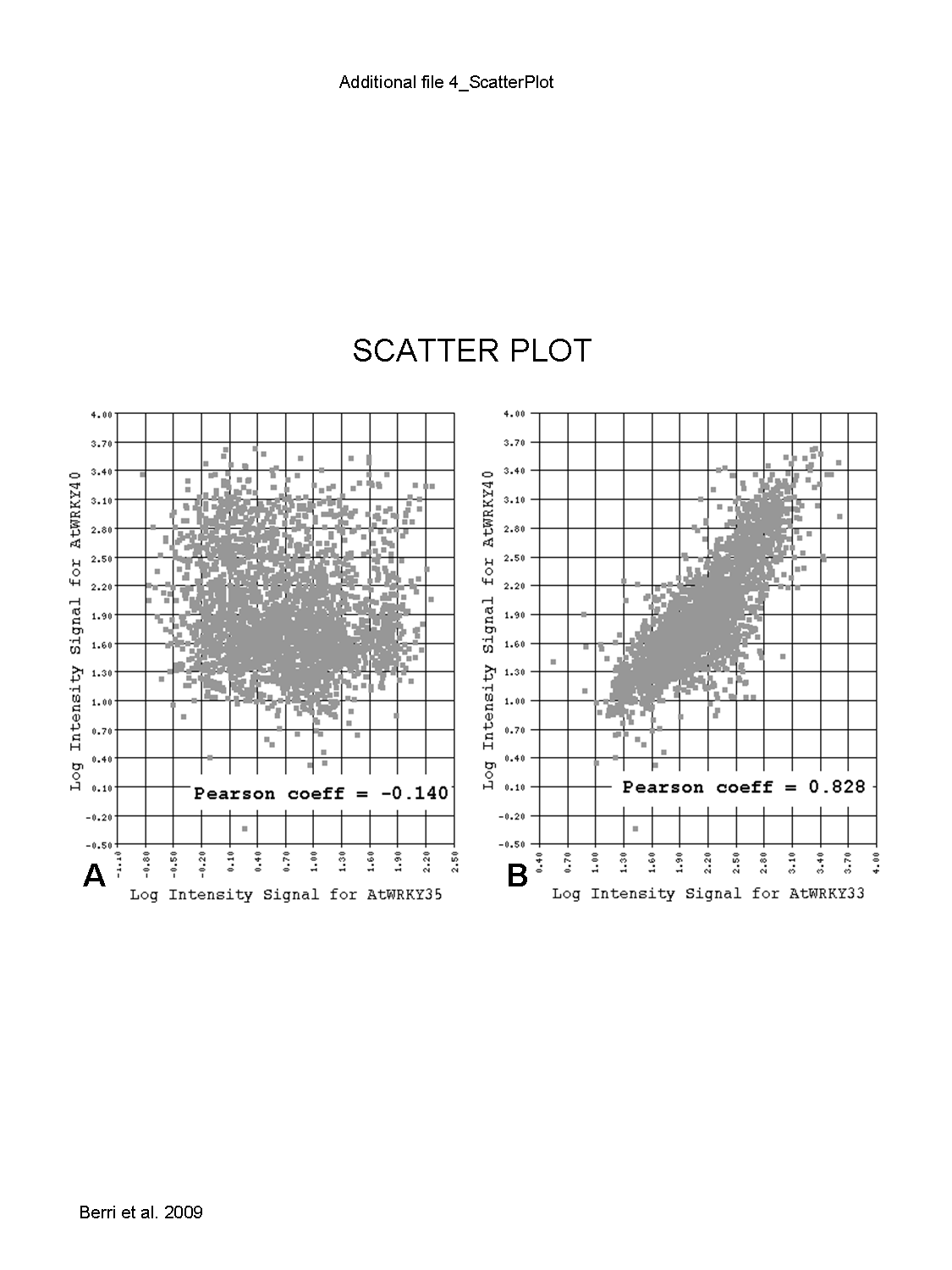

Supplement: Additional file 4 — AtWRKY genes scatter plots. Typical scatter plot of the expression level of two pairs of AtWRKY genes across the set of Arabidopsis microarray experiments used for the Pearson Correlation Coefficient analysis. Each grey dot represents the simultaneous expression level of the two genes in one microarray experiment. A: The expression level of AtWRKY40 is not correlated with the expression of AtWRKY35. B: A strong correlation is present between AtWRKY40 and AtWRKY33. [file 1471-2229-9-120-S4.png]

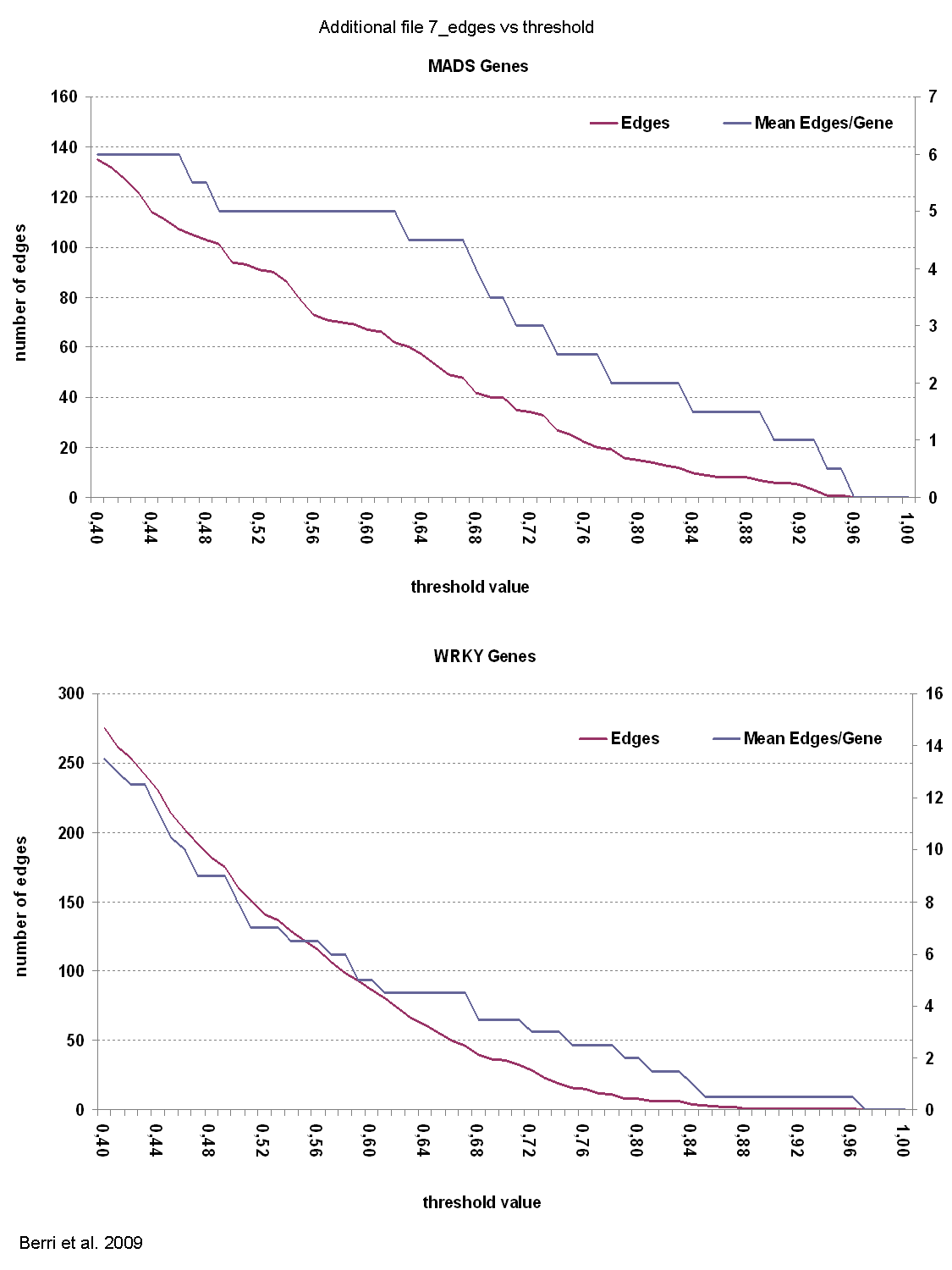

Supplement: Additional file 7 — Plot of edges and mean of edges/gene vs PCC threshold value. Plots of the number of edges (Y axis on the left) and mean of edges/gene (Y axis on the right) as a function of the PCC threshold values in the linear Pearson Correlation Coefficient analysis (P-lin) of the Arabidopsis MADS-BOX (above) and WRKY (below) genes. [file 1471-2229-9-120-S7.png]

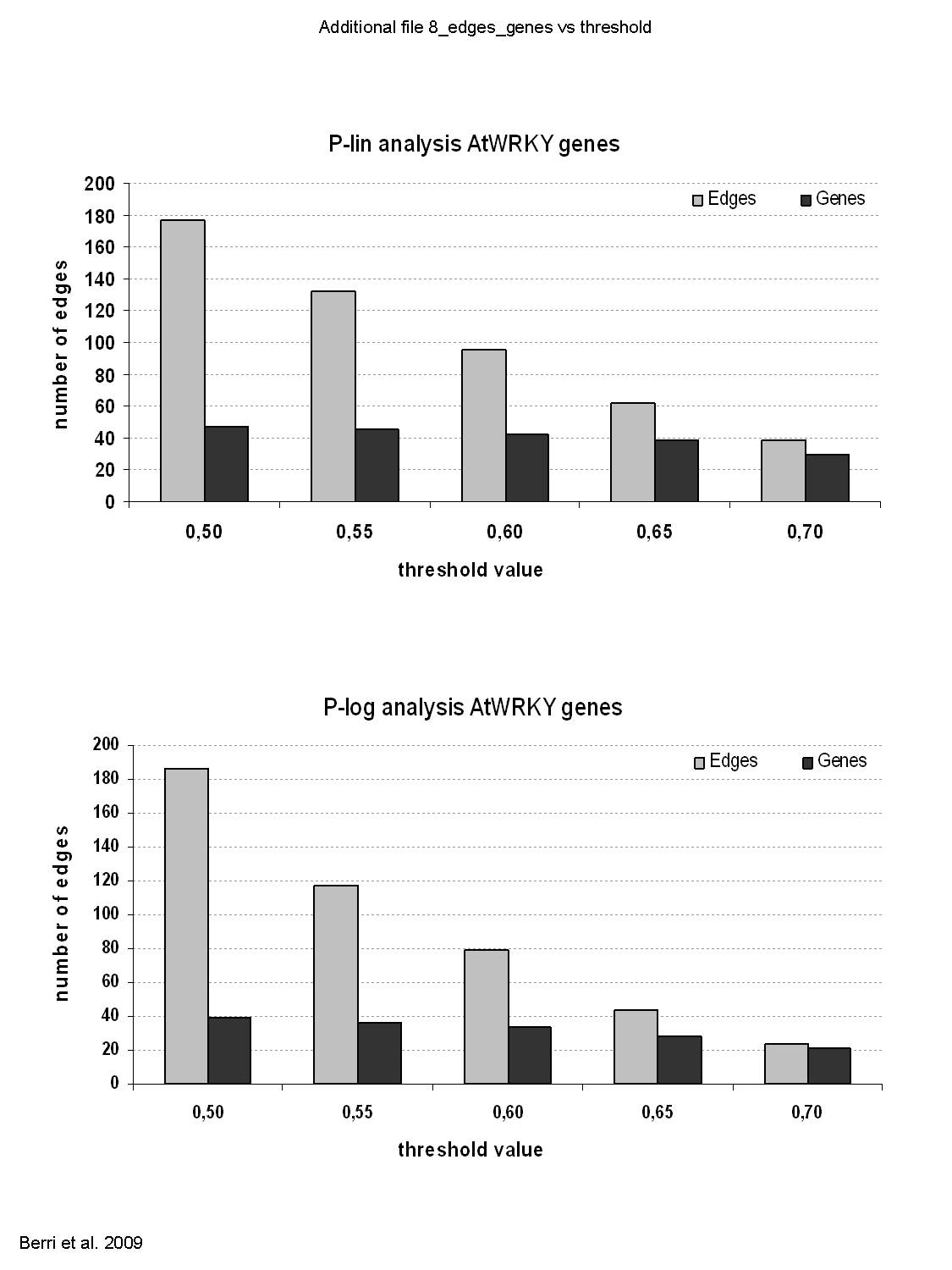

Supplement: Additional file 8 — Plot of edges and number of genes vs PCC threshold value. Plots of the number of edges and number of genes as a function of the PCC threshold values in the P-lin linear (above) and log-transformed (below) Pearson Correlation Coefficient analysis of the Arabidopsis WRKY (AtWRKY) genes. [file 1471-2229-9-120-S8.png]

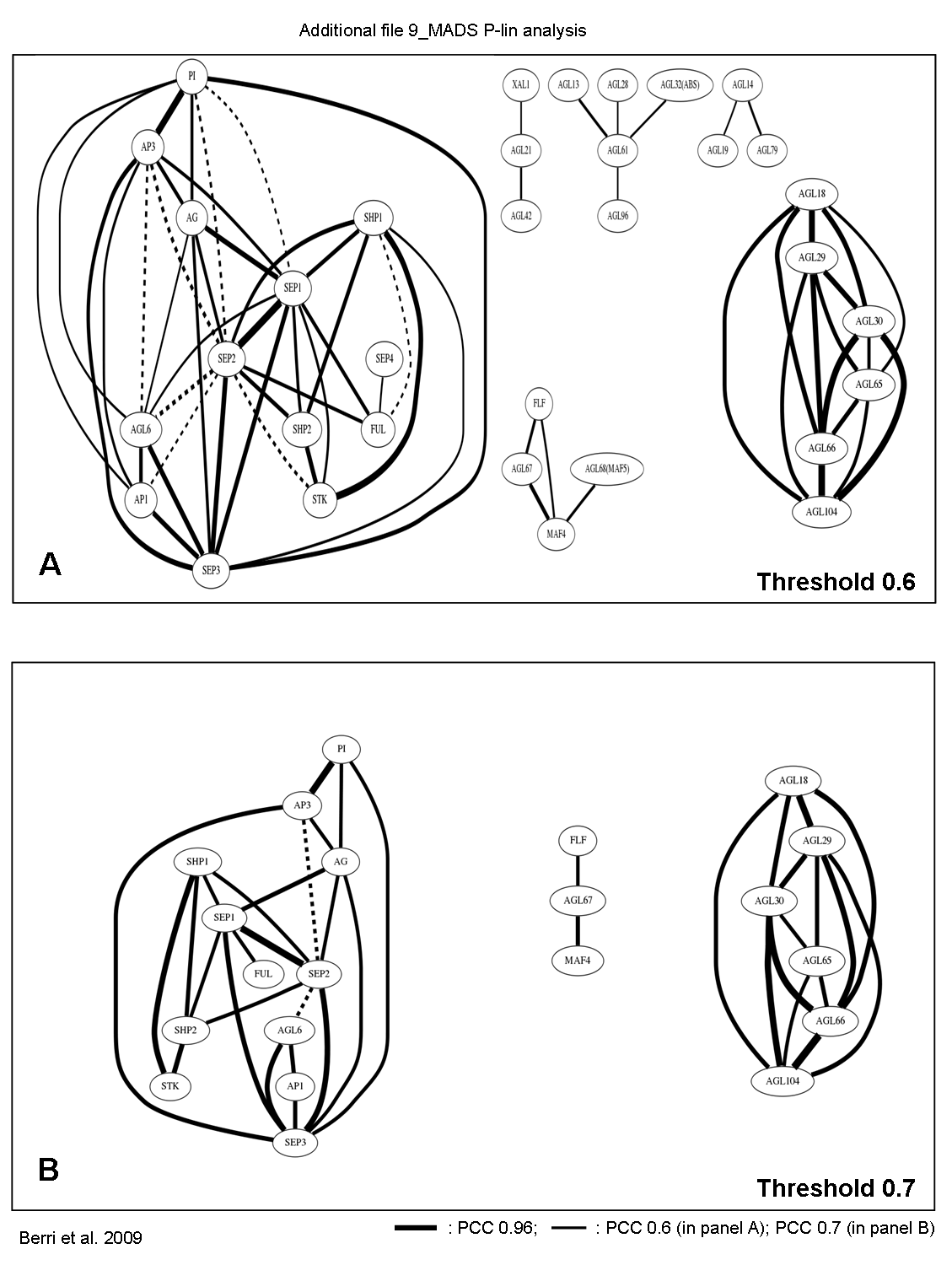

Supplement: Additional file 9 — P-lin co-regulatory networks of Arabidopsis MADS-BOX genes. Co-regulatory networks of Arabidopsis MADS-BOX genes obtained using untransformed Pearson Correlation Coefficient analysis (P-lin analysis). A: PCC threshold value of 0.6 B: PCC threshold value of 0.7. Unbroken lines indicate experimentally validated edges reported in literature; broken lines indicate edges not yet experimentally validated. The thickness of the edges is proportional to the value of the Pearson Coefficient. Thick black line: Pearson Correlation Coefficient 0.96; Thin Black Line: Pearson Correlation Coefficient 0.6 (in panel A) and 0.7 (in Panel B); the proximity of two genes on the graph is not indicative of their relatedness. [file 1471-2229-9-120-S9.png]

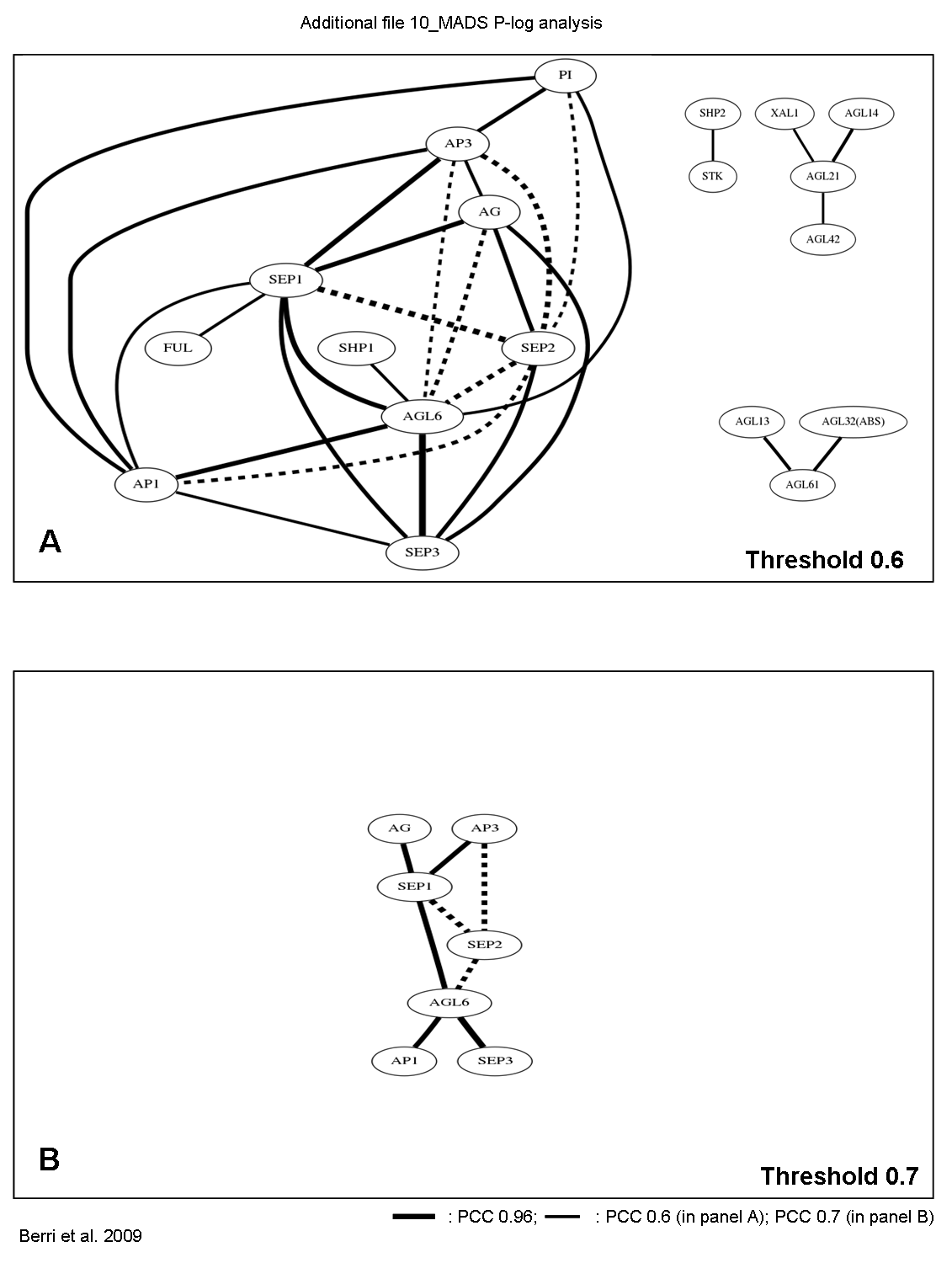

Supplement: Additional file 10 — P-log co-regulatory networks of Arabidopsis MADS-BOX genes. Co-regulatory networks of Arabidopsis MADS-BOX genes obtained using log-transformed Pearson Correlation Coefficient analysis (P-log analysis). A: PCC threshold value of 0.6 B: PCC threshold value of 0.7. Unbroken lines indicate experimentally validated edges reported in literature; broken lines indicate edges not yet experimentally validated. The thickness of the edges is proportional to the value of the Pearson Coefficient. Thick black line: Pearson Correlation Coefficient 0.96; Thin Black Line: Pearson Correlation Coefficient 0.6 (in panel A) and 0.7 (in Panel B); the proximity of two genes on the graph is not indicative of their relatedness. [file 1471-2229-9-120-S10.png]

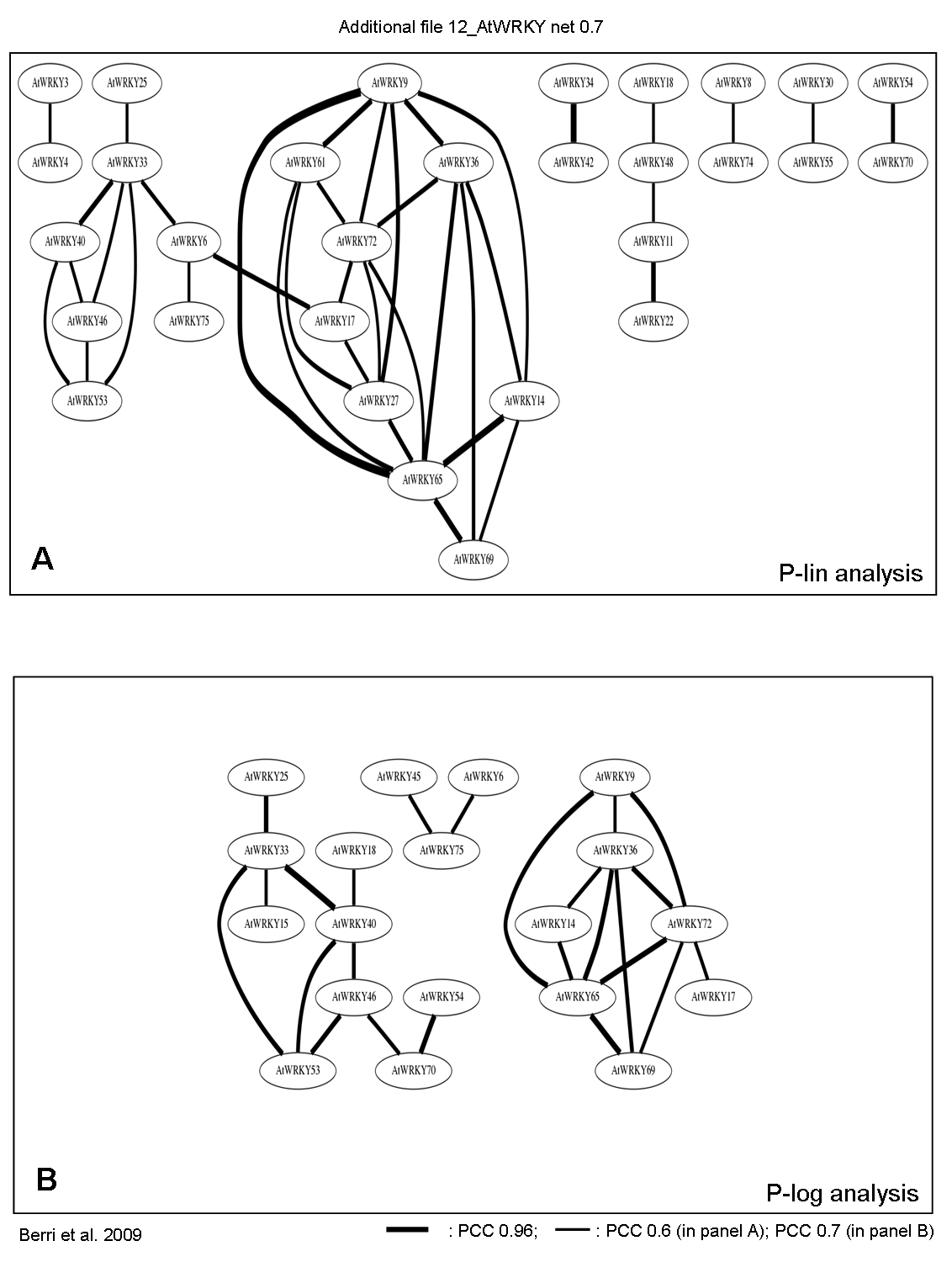

Supplement: Additional file 12 — Co-regulatory networks of Arabidopsis WRKY genes. Co-regulatory networks of Arabidopsis WRKY genes obtained with the PCC threshold value of 0.7 in the untransformed (P-lin) (panel A) and log transformed (P-log) Pearson Correlation Coefficient analysis (panel B). The thickness of the edges is proportional to the value of the Pearson Correlation Coefficient. Thick black line: Pearson Correlation Coefficient 0.96; Thin Black Line: Pearson Correlation Coefficient 0.7. The proximity of two genes on the graph is not indicative of their relatedness. [file 1471-2229-9-120-S12.png]
